# Autism risk following antidepressant medication during pregnancy

**DOI:** 10.1017/S0033291717001301

**Published:** 2017-05-22

**Authors:** A. Viktorin, R. Uher, A. Reichenberg, S. Z. Levine, S. Sandin

**Affiliations:** 1 Department of Psychiatry, Icahn School of Medicine, Mount Sinai, New York, NY, USA; 2 The Seaver Autism Center, Research and Treatment at Mount Sinai, New York, NY, USA; 3 Department of Medical Epidemiology and Biostatistics, Karolinska Institutet, Stockholm, Sweden; 4 Department of Psychiatry, Dalhousie University, Halifax, Nova Scotia, Canada; 5 Department of Community Mental Health, University of Haifa, Haifa, Israel

**Keywords:** Antidepressant, autism, depression, pregnancy, SSRI

## Abstract

**Background.:**

Previous studies have examined if maternal antidepressant medication during pregnancy increase the risk of autism spectrum disorder (ASD) in the offspring, but the results have been conflicting.

**Methods.:**

In a population-based cohort of 179 007 children born in 2006 and 2007 and followed through 2014 when aged 7 and 8, we estimated relative risks (RRs) of ASD and 95% confidence intervals (CIs) from Cox regression in children exposed to any antidepressant medication during pregnancy, and nine specific antidepressant drugs. Analyses were adjusted for potential confounders and were conducted in the full population sample, and in a clinically relevant sub-sample of mothers with at least one diagnosis of depression or anxiety during life.

**Results.:**

The adjusted RR of ASD in children of mothers who used antidepressant medication during pregnancy was estimated at 1.23 (95% CI 0.96–1.57), and at 1.07 (95% CI 0.80–1.43) in women with a history of depression or anxiety. Analyses of specific antidepressants initially revealed increased RRs of offspring ASD confined to citalopram and escitalopram (RR: 1.47; 95% CI 0.92–2.35) and clomipramine (RR: 2.86; 95% CI 1.04–7.82).

**Conclusion.:**

Medication with antidepressants during pregnancy does not appear to be causally associated with an increased risk of ASD in the offspring. Instead, the results suggest that the association is explained by factors related to the underlying susceptibility to psychiatric disorders. Based on these findings, the risk of ASD in the offspring should not be a consideration to withhold treatment with commonly used antidepressant drugs from pregnant women.

## Introduction

It has been suggested that antidepressant medication in pregnant women may increase the risk of autism spectrum disorder (ASD) in the offspring. This hypothesis has been examined in several studies, yet the results have been mixed; some studies have observed an increased risk (Croen *et al*. 2011; Rai *et al*. 2013; Gidaya *et al*. 2014; Harrington *et al*. 2014; Boukhris *et al*. 2016), and others have not (Hviid *et al*. 2013; Sorensen *et al*. 2013; Clements *et al*. 2015; Castro *et al*. 2016; Malm *et al*. 2016).

Pharmacotherapy plays a central role in the management of the depressive illness, and untreated depression has been associated with poor health outcomes in both mothers and offspring (Lecrubier, 2001; Marmorstein *et al*. 2004; O’Hara & McCabe, 2013; Meltzer-Brody & Stuebe, 2014). With the introduction of selective serotonin re-uptake inhibitors (SSRIs), the last two decades have seen a substantial increase in prescriptions of antidepressant drugs (Olfson & Marcus, 2009), also in pregnant women (Andrade *et al*. 2008; Bakker *et al*. 2008). Although SSRI antidepressants are typically well tolerated (Cipriani *et al*. 2009), the risk of adverse effects on the developing fetus has not been resolved.

An association between maternal antidepressant medication during pregnancy and offspring ASD could either be due to: (1) a direct effect of the drug or (2) factors that confound the antidepressant treatment and the outcome studied.

Previous reported associations between maternal antidepressant medication during pregnancy and ASD in the offspring have been limited to studies of antidepressant medications as a whole (Croen *et al*. 2011; Rai *et al*. 2013; Sorensen *et al*. 2013; Clements *et al*. 2015; Castro *et al*. 2016), or SSRI antidepressants as a whole (Hviid *et al*. 2013; Gidaya *et al*. 2014; Harrington *et al*. 2014; Boukhris *et al*. 2016; Malm *et al*. 2016). The risk of offspring ASD associated with specific antidepressant drugs is still unknown. If the risk is confined to certain drugs, the proportion of mothers treated with these drugs in prior studies may explain the varying results. Furthermore, recent studies indicate that psychiatric disorders share genetic determinants (International Schizophrenia Consortium *et al*. 2009; Lichtenstein *et al*. 2009; Sullivan *et al*. 2012; Jokiranta *et al*. 2013). Pregnant women suffering from a mental illness during pregnancy, for which antidepressants may be prescribed, likely carry genetic susceptibility that could be inherited by the child (Jokiranta *et al*. 2013). As such, the genetic susceptibility, rather than the medication, may completely or partially explain the increased risk of ASD in children of mothers using antidepressants during pregnancy. Yet, previous studies have not thoroughly examined to what extent the risk of offspring ASD may be influenced by underlying susceptibility to mental illness.

The aim of the study was to examine the association between maternal antidepressant medication during pregnancy and ASD in the offspring, investigating any antidepressant medication, and specific anti-depressant drugs. We performed an analysis using one of the most comprehensive databases for offspring ASD and parental disorder and medication data available. This allowed us to investigate specific antidepressant drugs used during pregnancy, and use detailed adjustment for parental psychiatric diagnoses. Moreover, we investigated both the full cohort and a clinically relevant sub-sample of mothers with any diagnosis of depression and/or anxiety in their lifetime.

## Methods

### Population

A birth cohort based on all live-born children conceived from July 1, 2005 and born in 2006 and 2007 was established by linkage of Swedish National registers using the unique individual Swedish national registration number (Ludvigsson *et al*. 2009). Offspring and mothers were identified in the Swedish Medical Birth Register that covers 99% of all births nation-wide since 1973 and provides information on gestational age at birth that were used to calculate the beginning of pregnancy (Cnattingius *et al*. 1990). In Sweden, 95% of all pregnant women receive early second trimester ultrasonography, which provide the gestational age of the fetus with an error margin of ±7 days (Hogberg & Larsson, 1997). The fathers were identified using the Multi-Generation Register (Ekbom, 2011). To be included, the children had to have complete information on gestational age at birth and the identity of the father. The study was approved by the Regional Ethics Committee in Stockholm, Sweden.

### Exposures

The Swedish Prescribed Drug Register holds information on all dispensed prescription drugs in Sweden since July 1, 2005 along with drug name, prescription-and dispensation dates, and the Anatomical Therapeutic Chemical Classification System (ATC) code (WHO, 2006; Wettermark *et al*. 2007). We identified dispensations of all psychotropic drugs prescribed in Sweden, including antidepressants, anxiolytics, stimulants, mood stabilizers, antipsychotics, and sedatives (online Supplementary Tables S1 and S2). The offspring were classified as unex-posed to antidepressants if they were born to mothers without any dispensation of an antidepressant with a medication period overlapping the pregnancy. Since the number of antidepressant dispensations was reduced during pregnancy, compared with prior or after (online Supplementary Fig. S1), it was assumed that a single anti-depressant dispensation overlapping pregnancy could represent medication that was halted prior the pregnancy. Therefore, antidepressant-exposed offspring were grouped into those born to a mother with either: (1) one single medication dispensation overlapping the pregnancy or (2) two or more dispensations of antidepressants with medication periods overlapping pregnancy (online Supplementary Fig. S2).

### Ascertainment of ASD

The children were followed from birth through 2014 when aged 7 or 8. A clinically ascertained diagnosis of offspring ASD was identified in the Swedish Patient Register (Ludvigsson *et al*. 2011; Sandin *et al*. 2016). This register includes all inpatient psychiatric admissions since 1973 and all outpatient specialist admissions since 2001, and provides admission dates along with the main and eight secondary diagnosis codes in accordance with the International Classification of Disease (ICD). Autism spectrum disorder was defined by having at least one in- or out-patient specialist care admission between birth and end of follow-up at December 31, 2014 with an ICD-10 code according to: F84.0, F84.1, F84.2, F84.3, F84.4, F84.5, F84.8, or F84.9. The ASD diagnoses in the Swedish Patient Register has previously been validated (Idring *et al*. 2012), and published on extensively (Sandin *et al*. 2016).

### Covariates

To adjust for potential temporal trends, the birth date of the offspring was included as number of days from January 1, 2005 to the birth date of the child. Maternal and paternal age at childbirth was categorized into below 20, 20–29, 30–39, and above 40 years of age. Maternal and paternal susceptibility to mental illness were ascertained based on having at least one psychiatric diagnosis in the Swedish Patient Register within several psychiatric disorder sub-groups at any time in life (online Supplementary Table S3). The father’s medication with any psychotropic drugs overlapping the pregnancy was also included, as well as mother’s dispensations of other psychotropic medication that overlapped the pregnancy.

### Statistical analysis

Relative risks (RRs) of ASD and the associated Wald-type two-sided 95% confidence intervals (CIs) were estimated by the hazard ratios from Cox regression models. The Cox regression models were fitted using days since birth as the underlying time scale. Each child was followed from birth until a diagnosis of ASD, death as identified in the Statistics Sweden register of vital statistics, or end of follow-up at December 31, 2014 – whichever came first. The RR of ASD was calculated in: (1) offspring born to mothers with a single-drug dispensation with a medication period overlapping the pregnancy and (2) offspring born to mothers with at least two dispensations with medication periods overlapping the pregnancy, compared with offspring born to mothers without a dispensation with a medication period overlapping the pregnancy.

Analyses were conducted in: (A) the complete sample to provide a public health perspective and in (B) a clinically relevant sub-sample of children born to mothers with at least one diagnosis of depression or anxiety in their lifetime, since most individuals who receive treatment with antidepressants also suffer from those disorders. In this sub-sample, the control group corresponds to mothers who are more likely to be considered for anti-depressant treatment, and may share a similar genetic susceptibility of mental illness as the medicated mothers.

First, we examined children of mothers medicated during pregnancy with any type of antidepressant. The RRs of ASD were calculated in a sequence of models with increasing degree of adjustment for potential confounding factors according to: model 1 crude analyses without covariate adjustment; model 2 analyses adjusted for including offspring birthdate, mother’s and father’s dispenses of other psychotropic medications during pregnancy, and maternal and paternal age; model 3 analyses with additional adjustment for any depressive diagnosis in the mother’s life time; and model 4 analyses further adjusted for any diagnosis of specific psychiatric disorder sub-groups in either the mother and/or father’s life time. The psychiatric disorder sub-groups included depression, anxiety disorders, schizophrenia, bipolar disorder, substance use disorder, compulsive disorder, attention-deficit hyperactive disorder, ASD, intellectual disability, and any other psychiatric diagnosis (online Supplementary Table S3).

Secondly, we further calculated the RR of ASD among children of mothers treated exclusively with one of the nine most prevalent antidepressants in the sample: sertra-line, citalopram and escitalopram, fluoxetine, venlafaxine, paroxetine, clomipramine, amitriptyline and nortriptyline, duloxetine, and mirtazapine, compared with children of mothers not treated with antidepressants during pregnancy. These analyses were adjusted for all included covariates, corresponding to model 4 in the analyses of any antidepressant. For the analyses examining specific drugs, to protect against an inflated error rate as a result of performing many statistical tests, we additionally present multiplicity-corrected *p* values using the Bonferroni–Holm procedure (Holm, 1979).

All tests of statistically significance were done at the nominal 5% level of significance. Data management and statistical analyses were done using SAS 9.4 and STATA/IC 14, respectively.

### Sensitivity analyses

The proportional hazards assumption for the Cox regression models was examined using Schoenfeld residuals (Grambsch & Therneau, 1994). To account for potential within-family correlations in the data due to multiple births from the same parents, we used bootstrap techniques (Efron & Tibshirani, 1994). The analyses were separately repeated for the outcome autistic disorder (ICD-10 F84.0). Potential sex specificity of associations was tested by analyses of male and female off-spring separately. Analyses were also conducted separately for SSRI antidepressants, non-SSRI antidepressants, and non-antidepressant psychotropic drugs. The role of socioeconomic status was examined by including an analysis adjusted for education length as a potential confounder. To further examine the effect of the underlying disorder susceptibility, we compared: (a) children born to mothers treated with antidepressants during pregnancy (*N* = 3982), to (b) children born to mothers with no psychotropic medication but diagnosed with: (1) at least one of type psychiatric disorder, (2) at least two psychiatric disorders, or (3) at least three psychiatric disorders according to online Supplementary Table S3.

## Results

Table 1 presents descriptive data for the cohort. Among the 180 444 children conceived from July 1, 2005 and born up until December 31, 2007 in the Medical Birth Register, 1437 (0.7%) did not have complete data and were excluded from the statistical analyses. Table 1 presents descriptive statistics for the 179 007 included children and their parents. At least one diagnosis of any psychiatric illness was observed in 47 204 (13.2%) parents. Autism spectrum disorder was observed in 1641 (0.9%) of the children, and among those, 1004 (61.0%) had a diagnosis of autistic disorder. Among the offspring, 2379 (1.3%) were born to a mother with a single antidepressant dispensation with a medication period overlapping pregnancy, and 3982 (2.2%) were born to a mother with at least two antidepressant dispensations with medication periods overlapping pregnancy.

Due to the ambiguity of drug exposure in children to mothers with only a single antidepressant dispensation overlapping pregnancy, the results focus on the children with at least two dispensations overlapping pregnancy, as compared with children without exposure to antidepressants. Results in the offspring of mothers with only a single dispensation overlapping pregnancy are presented in the online supplement (online Supplementary Fig. S3).

### Risk of ASD

In the full population sample, the crude RR of ASD in children of mothers with at least two dispensations of antidepressants overlapping pregnancy compared with unexposed children was estimated at 2.46 (95% CI 1.97–3.05). With adjustment for all included potential confounders, the RR was reduced to 1.23 (95% CI 0.96–1.57). In analyses confined to children of mothers with at least one diagnosis of depression or anxiety in their lifetime, the crude RR of ASD in children of mothers with at least two dispensations of antidepressants overlapping pregnancy was estimated at 1.30 (95% CI 0.99–1.71). With adjustment for potential confounders, the RR was reduced to 1.07 (0.80–1.43) (Table 2).

### Risk of ASD associated with specific medications

In the full population sample, the adjusted RRs of ASD in children of mothers with at least two dispensations of specific antidepressants overlapping pregnancy compared with unexposed children was estimated at 1.35 (95% CI 0.87–2.08) for sertraline, 1.71 (95% CI 1.16–2.51) for citalopram and escitalopram, 1.04 (95% CI 0.53–2.02) for fluoxetine, 1.22 (95% CI 0.54–2.75) for venlafaxine, 1.40 (95% CI 0.52–3.76) for paroxetine, 3.27 (95% CI 1.33–8.00) for clomipramine, 0.59 (95% CI 0.08–4.20) for amitriptyline and nortriptyline, and 1.53 (95% CI 0.38–6.23) for mirtazapine (Table 3).

Within the sub-sample where both the medicated and non-medicated women had at least one diagnosis of depression or anxiety in their lifetime, the adjusted RRs of ASD in children of mothers with at least two dispensations of specific antidepressants overlapping pregnancy compared with unexposed children was estimated at 1.17 (95% CI 0.71–1.95) for sertraline, 1.47 (95% CI 0.92–2.35) for citalopram and escitalopram, 1.08 (95% CI 0.53–2.21) for fluoxetine, 0.88 (95% CI 0.32–2.38) for venlafaxine, 1.21 (95% CI 0.38–3.80) for paroxetine, 2.86 (95% CI 1.04–7.82) for clomipramine, and 1.00 (95% CI 0.14–7.24) for mirtaza-pine (Table 3).

None of the analyses examining specific drugs, in the full sample and in the clinically relevant sub-sample, revealed statistically significant *p* values after multiplicity correction using the Bonferroni–Holm procedure (Holm, 1979).

### Sensitivity analyses

Inspection of the Schonfeld residuals did not suggest any violation of the proportional hazards assumption (online Supplementary Fig. S4). Analyses confined to specifically SSRI antidepressants, non-SSRI antidepressants, and non-antidepressant psychotropic drugs revealed results quantitatively similar to the analyses of any antidepressant (online Supplementary Fig. S5). Confidence intervals of selected point estimates (RRs) calculated using bootstrap revealed close to identical results as the parametric Wald estimates (online Supplementary Fig. S6). Analyses confined to autistic disorder showed results similar to that of ASD (online Supplementary Fig. S7). Analyses of male and female offspring separately did not reveal any sex-specific association (online Supplementary Fig. S8). The complementary analysis adjusting for education as a marker of socioeconomic status did not affect our results (online Supplementary Fig. S9). Analyses with comparison groups without psychotropic medication, but with increasing number of diagnosed psychiatric diagnoses revealed that the RR of ASD in the offspring was closely correlated with the number of different psychiatric disorders diagnosed in the mothers (online Supplementary Figs. S11 and S12).

## Discussion

In this population-based, prospective cohort study of 179 007 children and their parents, we observed an increased RR of ASD in offspring of mothers treated with antidepressant medication during pregnancy compared with offspring of mothers not treated with antidepressants during pregnancy. However, detailed adjustments for confounding by parental psychiatric liability attenuated this risk. Moreover, when the analyses were restricted to mothers ever diagnosed with depression or anxiety, a likely target group for anti-depressant medication, the association between antidepressants in pregnancy and ASD were attenuated even further and close to none.

To our knowledge, this is the first study to examine the RR of offspring ASD associated with specific types of antidepressant drugs compared with children unex-posed to antidepressants. Among the nine studied drugs, only the SSRI antidepressants citalopram and escitalopram, and the tricyclic antidepressant clomipramine displayed an increased RR of ASD in the off-spring. While these findings could suggest that these particular medications may have a causal effect associated with increased risk of ASD in the offspring, these positive associations could also be due to residual confounding. Citalopram and escitalopram have similar mechanisms of action as sertraline and paroxetine, yet only citalopram and citalopram displayed an elevated RR of ASD in the offspring. Furthermore, this increase in RR was modest, not statistically significant in the clinically relevant sub-sample, and not statistically different from the results of the other specific antidepressants studied. The results of the present study strongly suggest that the associations between antidepressant treatment during pregnancy and offspring ASD are gradually attenuated with increasing covariate adjustment. Although the present study adjusts for a broad set of factors that may confound the association, the ability to capture confounding is not complete. Mothers treated with different antidepressant medications may be different in other aspects that the current study cannot adjust for. This is exemplified by clomipramine that is not recommended as a first line of treatment of depression, which therefor may be given to women with a more severe or complex form of depression. Further analysis of this treatment group indeed revealed an increased prevalence of both compulsive disorder and schizophrenia compared with women treated with other antidepressants (online Supplementary Fig. S10).

To further investigate the role of psychiatric disorders in the association between antidepressant treatment during pregnancy and ASD in the offspring, we compared children exposed to antidepressants to children of mothers not using psychotropic medication during pregnancy, but with increasing mean number of psychiatric disorders (online Supplementary Fig. S11). These analyses revealed that a higher mean number of different psychiatric disorders diagnosed during the mothers’ lifetime was correlated with the RR of ASD in the offspring. This was also observed in analyses of specific antidepressant drugs (online Supplementary Fig. S12)

Nevertheless, the relationship between treatment with specific antidepressants during pregnancy and the risk of ASD in the offspring should be examined in additional samples to shed more light on the association and the specific underlying disorders.

Prior research into the association between maternal antidepressant medication during pregnancy and off-spring ASD has delivered mixed and inconclusive results (Croen *et al*. 2011; Hviid *et al*. 2013; Rai *et al*. 2013; Sorensen *et al*. 2013; Gidaya *et al*. 2014; Harrington *et al*. 2014; Clements *et al*. 2015; Boukhris *et al*. 2016; Castro *et al*. 2016; Malm *et al*. 2016). The present findings may resolve this earlier ambiguity. Our findings of attenuated associations with incremental covariate adjustment are in line with several earlier studies (Hviid *et al*. 2013; Sorensen *et al*. 2013; Clements *et al*. 2015; Castro *et al*. 2016; Malm *et al*. 2016). The present findings are, however, at odds with number of previous studies that report statistically sign-ificant associations. A study of 1054 Canadian children with ASD (Boukhris *et al*. 2016) reported an unadjusted RR of ASD following SSRI treatment (RR = 2.27; 95% CI 1.48–3.46) that were partially attenuated after adjustments for factors, including maternal history of depression (RR = 1.75; 95% CI 1.03–2.97). Similarly sized RRs have also been reported in a Californian sample of 298 children with ASD (RR = 2.2; 95% CI 1.2–4.3) (Croen *et al*. 2011), in a Danish sample of 5215 children with ASD (adjusted RR = 1.8; 95% CI 1.4–2.3) (Gidaya *et al*. 2014), in a US study of 421 male cases with ASD (adjusted RR = 2.91; 95% CI 1.07–7.93) (Harrington *et al*. 2014), and in a Swedish study of 4429 cases with ASD (RR = 3.34; 95% CI 1.50–7.47) (Rai *et al*. 2013).

The discrepancies between the results of previous studies and the present study could be due to several factors. If the present findings of different psychiatric disorder profiles in mothers treated with specific anti-depressants (online Supplementary Figs. S11 and S12) was comparable in previous studies, the proportion of these specific antidepressant drugs could explain the mixed results in previous studies. However, the limited adjustment for the parents’ mental illnesses in previous studies may also explain the discrepancies.

Strengths of the study include a large prospective population-based sample of children and their parents with close to complete healthcare data coverage. Any estimate of prevalence of ASD is highly dependent on the birth year, follow-up time, and sex distribution in the sample, still our estimates agree well with other studies in the Nordic countries and in the USA (Idring *et al*. 2012; Sandin *et al*. 2016). Data from a healthcare system with equal access limit the risk of selection biases. Inclusion of children born during a limited period reduces potential confounding by factors that may vary over time. Examination of any diagnosis of mental illness in the parents’ lifetime allowed detailed adjustment for confounding due to, e.g., genetic liability. The requirement of at least two dispensations overlapping the pregnancy further increased the specificity of the exposure. Furthermore, supplementary analyses of antidepressants divided into specifically SSRI anti-depressants and non-SSRI antidepressants, and nonantidepressant psychotropic drugs all revealed similar findings, lending further support to the interpretation that the mental illness confounds the association between maternal antidepressant medication during pregnancy and increased risk of ASD in offspring.

However, the findings should also be interpreted in light of some limitations. Overall, our study summarizes the results of a multitude of statistical tests, which always increase the risk of erroneous rejection of a specific hypothesis. When applying the Bonferroni–Holm procedure in the comparison of specific drugs compared with ‘no treatment of antidepressants’, no drugs were at a statistically significantly higher risk. Although drugs recorded in the Prescribed Drug Register have been prescribed and collected, we cannot be entirely sure to what extent the medication was actually consumed. To address this limitation, we divided the exposed children into those born to a mother with only a single dispensation, and those with at least two dispensations overlapping the pregnancy. The study focused on those with at least two dispensations, as a continuous dispensation pattern was assumed to better reflect an ongoing treatment. Although we adjusted for both parents’ education level at childbirth, we could not adjust for the income of the family. While education level had no effect on the association in our Swedish sample, this may not be the case in cohorts from other countries. Moreover, the Patient Register does not provide information from the primary care, and as such, any diagnoses confined to a primary care setting will go undetected. Still, this will not affect the study’s ability to detect children with an ASD, as they are referred to a specialist that is covered by the Patient Register. The lack of primary care data may, however, affect the study’s ability to capture psychiatric diagnoses as measure of mental illness among the parents, and consequently the ability to adjust for potential confounding due to underlying mental illness diagnosed in a primary care setting only. Antidepressant prescriptions are accompanied with a diagnosis in Sweden, yet supplemental analyses show that about 25% of mothers treated with an antidepressant during pregnancy lack a psychiatric diagnosis in the Patient Register (online Supplementary Fig. S13), which could indicate residual confounding. Moreover, the severity of the psychiatric disorders investigated was not known, nor if the mothers experienced ongoing episodes of the lifetime diagnosed disorders. It is likely that mothers treated with antidepressants during pregnancy experienced a more severe disorder than unmedicated mothers with a lifetime diagnosis of depression or anxiety. Finally, the current study cohort was restricted to children born in 2006 and 2007, and therefore is underpowered to perform family-control analyses, which can be valuable to address potential residual confounding due to shared familial and genetic factors.

## Conclusion

Medication with antidepressants during pregnancy does not appear to be causally associated with an increased risk of ASD in the offspring. Instead, the results suggest that the association is explained by factors related to the underlying susceptibility to psychiatric disorders. Based on these findings, the risk of ASD in the offspring should not be a consideration to withhold treatment with commonly used antidepressant from pregnant women.

## Supplementary Material

Supplement

## Figures and Tables

**Table 1.** Cohort subject characteristics

Characteristics, no (%)	Full population sample			Clinical sub-sample^a^		
	Antidepressant medication during pregnancy			Antidepressant medication during pregnancy		
	Unexposed^b^	Exposed^c^	Ambiguous^d^	Unexposed^b^	Exposed^c^	Ambiguous^d^
Number of offspring	172 646 (96.5)	3982 (2.2)	2379 (1.3)	14 805 (79.8)	2617 (14.1)	1129 (6.1)
Offspring diagnosed with autism spectrum disorder	1524 (0.9)	85 (2.1)	32 (1.4)	282 (1.9)	62 (2.4)	17 (1.5)
Offspring diagnosed with autistic disorder	942 (0.6)	41 (1.0)	21 (0.9)	164 (1.1)	29 (1.1)	11 (1.0)
Maternal use of psychotropic medication other than antidepressants during pregnancy	1297 (0.8)	595 (14.9)	143 (6.0)	423 (2.9)	478 (18.3)	97 (8.6)
Paternal psychotropic medication during pregnancy	5875 (3.4)	437 (11.0)	181 (7.6)	840 (5.7)	314 (12.0)	98 (8.7)
Birth year						
2006	73 002 (42.3)	1588 (39.9)	951 (40.0)	6381 (43.1)	1036 (39.6)	443 (39.2)
2007	99 644 (57.7)	2394 (60.1)	1428 (60.0)	8424 (56.9)	1581 (60.4)	686 (60.8)
Maternal psychiatric diagnosis	23 551 (13.6)	2868 (72.0)	1312 (55.2)	14 805 (100)	2617 (100)	1129 (100)
Paternal psychiatric diagnosis	18 201 (10.5)	836 (21.0)	436 (18.3)	3056 (20.6)	623 (23.8)	258 (22.9)
Maternal age (years) at delivery						
<20	3367 (2.0)	61 (1.5)	59 (2.5)	706 (4.8)	48 (1.8)	47 (4.2)
20–29	73 009 (42.3)	1585 (39.8)	975 (41.0)	7239 (48.9)	1119 (42.8)	505 (44.7)
30–39	91 716 (52.5)	2123 (53.3)	1235 (51.9)	6374 (43.1)	1316 (50.3)	532 (47.1)
40+	5554 (3.2)	213 (5.35)	110 (4.6)	486 (3.3)	134 (5.1)	45 (4.0)
Paternal age (years) at delivery						
<20	984 (0.6)	21 (0.5)	17 (0.7)	246 (1.7)	15 (0.6)	12 (1.1)
20–29	47 994 (27.8)	1163 (29.2)	743 (31.2)	5337 (36.1)	840 (32.1)	406 (36.0)
30–39	100 624 (58.3)	2160 (54.2)	1259 (52.9)	7240 (48.9)	1337 (51.1)	568 (50.3)
40+	23 044 (13.4)	638 (16.0)	360 (15.1)	1982 (13.4)	425 (16.2)	143 (12.7)
Offspring sex						
Males	88 858 (51.5)	2043 (51.3)	1232 (51.8)	7517 (50.8)	1349 (51.5)	585 (51.8)
Females	83 788 (48.5)	1939 (48.7)	1147 (48.2)	7288 (49.2)	1268 (48.5)	544 (48.2)

a A clinically relevant sub-sample consisting of 18 551 children, of which 361 had been diagnosed with autism spectrum disorder. All mothers, both medicated and non-medicated, had at least one diagnosis of depression or an anxiety disorder in their lifetime (online Supplementary Table S3). Thereby, the offspring of mothers with medication during pregnancy is contrasted with offspring of mothers that may share similar underlying factors.

b Children of mothers with no antidepressant dispensation with a medication period overlapping pregnancy.

c Children of mothers with at least two antidepressant dispensations with medication periods overlapping pregnancy (online Supplementary Fig. S1 examples A1 and A2).

d Children of mothers with a single antidepressant dispensation with a medication period overlapping pregnancy.

**Table 2.** Relative risks of autism spectrum disorder due to exposure to any type of antidepressants^a^

	Full sample, *N =* 179 007	Clinical sub-sample^b^, *N* = 18 551	Forest plot
	Relative risk (95% CI)	Relative risk (95% CI)	
Model l^c^	2.46 (1.97–3.05)	1.30 (0.99–1.71)	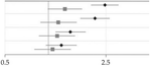
Model 2^d^	2.10 (1.66–2.65)	1.17 (0.88–1.57)	
Model 3^e^	1.42 (1.12–1.81)	1.15 (0.86–1.53)	
Model 4^f^	1.23 (0.96–1.57)	1.07 (0.80–1.43)	

ASD, autism spectrum disorder; CI, confidence interval.

a The sample consists of 179,007 children born during 2006 and 2007, of which 1,641 had been diagnosed with ASD. The figure presents relative risks of ASD and two-sided 95% confidence intervals in children of mothers with at least two dispensations of a specific antidepressant drug overlapping the pregnancy, compared with unexposed children. The analyses are adjusted for all included covariates, as in model 4 in Figure 1.

b The clinical sub-sample consists of 18 551 children, of which 361 had been diagnosed with autism spectrum disorder. All mothers, both medicated and non-medicated, had at least one diagnosis of depression or an anxiety disorder in their lifetime (online Supplementary Table S3). Thereby, the offspring of mothers with medication during pregnancy is contrasted with offspring of mothers that may share similar underlying factors.

c Analyses not adjusted for covariates.

d Analyses adjusted for birthdate, maternal and paternal age, the father’s psychotropic medication that overlapped the pregnancy, and the mother’s one-time dispensations of psychotropic medication that overlapped the pregnancy.

e Analyses adjusted for the factors listed in footnote ^d^, and for any diagnosis of depression in the mother’s lifetime (yes/no) (see online Supplementary Table S3 for specific diagnosis codes).

f Analyses adjusted for the factors listed in footnote ^d^, and for any diagnosis of specific psychiatric disorder sub-groups in either the mother and/or father’s life time (yes/no), including depression, anxiety disorders, substance use disorder, bipolar disorder, compulsive disorder, attention deficit hyperactivity disorder, autism spectrum disorder, intellectual disability, schizophrenia, and ‘other psychiatric diagnosis’ (see Supplementary Table S3 for specific diagnosis codes).

**Table 3.** Relative risks of autism spectrum disorder due to exposure to specific antidepressants^a^

	Full sample, *N=* 179 007					Clinical sub-sample^b^, *N*= 18 551					Forest plot
Medication	Exposed children	Exposed children with ASD (%)	Relative risk (95% CI)	*p* value	Bonferroni-Holm corrected *p* value^c^	Exposed children	Exposed children with ASD (%)	Relative risk (95% CI)	*p* value	Bonferroni-Holm corrected *p* value^c^	
Sertraline	1016	22 (2.2)	1.35 (0.87–2.08)	0.186	1.000	672	16 (2.4)	1.17 (0.71–1.95)	0.485	1.000	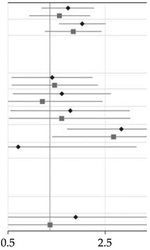
Citalopram and escitalopram	1097	28 (2.6)	1.71 (1.16–2.51)	0.006	0.090	639	19 (3.0)	1.47 (0.92–2.35)	0.100	1.000	
Fluoxetine	458	9 (2.0)	1.04 (0.53–2.02)	0.919	1.000	327	8 (2.4)	1.08 (0.53–2.21)	0.783	1.000	
Venlafaxine	266	6 (2.3)	1.22 (0.54–2.75)	0.632	1.000	195	4 (2.1)	0.88 (0.32–2.38)	0.819	1.000	
Paroxetine	173	4 (2.3)	1.40 (0.52–3.76)	0.516	1.000	108	3 (2.8)	1.21 (0.38–3.80)	0.714	1.000	
Clomipramine	91	5 (5.5)	3.27 (1.33–8.00)	0.010	0.140	68	4 (5.9)	2.86 (1.04–7.82)	0.034	0.442	
Amitriptyline and nortriptyline	133	1 (0.8)	0.59 (0.08–4.20)	0.597	1.000	40	0 (0)	*NA*	*NA*	*NA*	
Duloxetine	52	0 (0)	*NA*	*NA*	*Na*	37	0 (0)	*NA*	*NA*	*NA*	
Mirtazapine	62	2 (3.2)	1.53 (0.38–6.23)	0.376	1.000	36	1 (2.8)	1.00 (0.14–7.24)	1.000	1.000	

ASD, autism spectrum disorder; N, number of children; CI, confidence interval; NA, not available.

a The sample consists of 179 007 children born during 2006 and 2007, of which 1641 had been diagnosed with ASD. The figure presents relative risks of ASD and two-sided 95% CIs in children of mothers with at least two dispensations of a specific antidepressant drug overlapping the pregnancy, compared with unexposed children. The analyses are adjusted for all included covariates, as in model 4 in Table 2.

b The clinical sub-sample consists of 18 551 children, of which 361 had been diagnosed with ASD. All mothers, both medicated and non-medicated, had at least one diagnosis of depression or an anxiety disorder in their lifetime (online Supplementary Table S3). Thereby, the offspring of mothers with medication during pregnancy is contrasted with offspring of mothers that may share similar underlying factors.

c Multiplicity-corrected *p* values using the Bonferroni–Holm procedure: Holm (1979). A simple sequentially rejective multiple test procedure. *Scandinavian Journal of Statistics* 6, pp. 65–70.
