# Supplementary material for: Autism risk following antidepressant medication during pregnancy
Source: Psychol Med. Author manuscript; Available in PMC 2019 Mar 18. (PMC6421839; doi:10.1017/S0033291717001301)
Supplement: Supplement [file NIHMS1006623-supplement-Supplement.pdf]

SUPPLEMENTARY APPENDIX

This appendix has been provided by the authors to give readers additional information about their work.

Supplement to: Viktorin A, Uher R, Reichenberg A, Levine SZ, Sandin S. Antidepressant medication during pregnancy and autism spectrum disorder.

TABLE OF CONTENTS

Supplementary Table 1. Complete list of ATC codes and names of drugs considered in the study......................**page 2**

Supplementary Table 2. Psychotropic drugs with at least one dispensation overlapping the pregnancy..............**page 3**

Supplementary Figure 1. Antidepressant dispensations around pregnancy...**page 4**

Supplementary Figure 2. Medication exposure definition... **page 5**

Supplementary Table 3. Mental illness sub-groups and corresponding diagnosis codes.....................................**page 6**

Supplementary Figure 3. Relative risks of ASD in offspring of mothers with one, or at least two medication dispensations overlapping pregnancy..**page 7**

Supplementary Figure 4. Plotted Schoenfeldt residuals for each covariate with 4 degrees of freedom................**page 8**

Supplementary Figure 5. Relative risks of ASD in offspring of mothers treated with any antidepressant, or specifically SSRI antidepressants, non-SSRI antidepressants, or non-antidepressant psychotropic drugs...**page 13**

Supplementary Figure 6. Relative risks of ASD from bootstrapped analyses.. **page 14**

Supplementary Figure 7. Relative risks of autistic disorder ....**page 15**

Supplementary Figure 8. Sex-combined and sex-specific relative risks of ASD.. **page 16**

Supplementary Figure 9. Analysis additionally adjusted for socioeconomic status as parental education level... **page 17**

Supplementary Figure 10. Percentage of mothers with at least one lifetime diagnosis in different psychiatric disorder categories...**page 18**

Supplementary Figure 11. Relative risks of ASD in offspring of mothers treated with any antidepressant, compared to control children of mothers not using any psychotropic medication but with increasing mean number of psychiatric disorders diagnosed during lifetime.. **page 19**

Supplementary Figure 12. Relative risks of ASD in offspring of mothers treated with specific antidepressants, compared to control children of mothers not using any psychotropic medication but with increasing mean number of psychiatric disorders diagnosed during lifetime.. **page 20**

Supplementary Figure 13. Percentage of medicated mothers with a lifetime psychiatric diagnosis......................**page 21**

Supplementary Table 1. Complete list of ATC codes and names of drugs considered in the study

ATC	Drug name	ATC	Drug name	ATC	Drug name	ATC	Drug name	ATC	Drug name
N06AA01	Desipramine	N06AX08	Bifemelane	N05CD05	Triazolam	N05AC01	Periciazine	N05AX13	Paliperidone
N06AA02	Imipramine	N06AX09	Viloxazine	N05CD06	Lormetazepam	N05AC02	Thioridazine	N05AX14	Iloperidone
N06AA03	Imipramine Oxide	N06AX10	Oxaflozane	N05CD07	Temazepam	N05AC03	Mesoridazine	N05AX15	Cariprazine
N06AA04	Clomipramine	N06AX11	Mirtazapine	N05CD08	Midazolam	N05AC04	Pipotiazine	N05AX16	Brexpiprazole
N06AA05	Opipramol	N06AX12	Bupropion	N05CD09	Brotizolam	N05AD01	Haloperidol	N03AG01	Valproic Acid
N06AA06	Trimipramine	N06AX13	Medifoxamine	N05CD10	Quazepam	N05AD02	Trifluperidol	N03AX09	Lamotrigine
N06AA07	Lofepramine	N06AX14	Tianeptine	N05CD11	Loprazolam	N05AD03	Melperone	N06BA01	Amfetamine
N06AA08	Dibenzepin	N06AX15	Pivagabine	N05CD12	Doxefazepam	N05AD04	Moperone	N06BA02	Dexamfetamine
N06AA09	Amitriptyline	N06AX16	Venlafaxine	N05CD13	Cinolazepam	N05AD05	Pipamperone	N06BA03	Metamfetamine
N06AA10	Nortriptyline	N06AX17	Milnacipran	N03AE01	Clonazepam	N05AD06	Bromperidol	N06BA04	Methylphenidate
N06AA11	Protriptyline	N06AX18	Reboxetine	N05CF01	Zopiclone	N05AD07	Benperidol	N06BA05	Pemoline
N06AA12	Doxepin	N06AX19	Gepirone	N05CF02	Zolpidem	N05AD08	Droperidol	N06BA06	Fencamfamin
N06AA13	Iprindole	N06AX21	Duloxetine	N05CF03	Zaleplon	N05AD09	Fluanisone	N06BA07	Modafinil
N06AA14	Melitracen	N06AX22	Agomelatine	N05CF04	Eszopiclone	N05AE01	Oxypertine	N06BA08	Fenozolone
N06AA15	Butriptyline	N06AX23	Desvenlafaxine	N06BX07	Oxiracetam	N05AE02	Molindone	N06BA09	Atomoxetine
N06AA16	Dosulepin	N06AX24	Vilazodone	N06BX08	Pirisudanol	N05AE03	Sertindole	N06BA10	Fenetylline
N06AA17	Amoxapine	N06AX25	Hypericiherba	N06BX09	Linopirdine	N05AE04	Ziprasidone	N06BA11	Dexmethylphenidate
N06AA18	Dimetacrine	N06AX26	Vortioxetine	N06BX10	Nizofenone	N05AE05	Lurasidone	N06BA12	Lisdexamfetamine
N06AA19	Amineptine	N05BA01	Diazepam	N06BX11	Aniracetam	N05AF01	Flupentixol	N06BA13	Armodafinil
N06AA21	Maprotiline	N05BA02	Chlordiazepoxide	N06BX12	Acetylcarnitine	N05AF02	Clopenthixol	N06BX01	Meclofenoxate
N06AA23	Quinupramine	N05BA03	Medazepam	N06BX13	Idebenone	N05AF03	Chlorprothixene	N06BX02	Pyritinol
N06AB02	Zimelidine	N05BA04	Oxazepam	N06BX14	Prolintane	N05AF04	Tiotixene	N06BX03	Piracetam
N06AB03	Fluoxetine	N05BA05	Potas. Clorazepate	N06BX15	Pipradrol	N05AF05	Zuclopenthixol	N06BX04	Deanol
N06AB04	Citalopram	N05BA06	Lorazepam	N06BX16	Pramiracetam	N05AG01	Fluspirilene	N06BX05	Fipexide
N06AB05	Paroxetine	N05BA07	Adinazolam	N06BX17	Adrafinil	N05AG02	Pimozide	N06BX06	Citicoline
N06AB06	Sertraline	N05BA08	Bromazepam	N06BX18	Vinpocetine	N05AG03	Penfluridol	A03CA01	Isopropamide And Psycholeptics
N06AB07	Alaproclate	N05BA09	Clobazam	N06BX21	Mebicar	N05AH01	Loxapine	A03CA02	Clidinium And Psycholeptics
N06AB08	Fluvoxamine	N05BA10	Ketazolam	N06BX22	Phenibut	N05AH02	Clozapine	A03CA03	Oxyphencyclimine And Psycholeptics
N06AB09	Etoperidone	N05BA11	Prazepam	N05AA01	Chlorpromazine	N05AH03	Olanzapine	A03CA04	Otilonium Bromide And Psycholeptics
N06AB10	Escitalopram	N05BA12	Alprazolam	N05AA02	Levomepromazine	N05AH04	Quetiapine	A03CA05	Glycopyrronium Bromide And Psycholeptics
N06AF01	Isocarboxazid	N05BA13	Halazepam	N05AA03	Promazine	N05AH05	Asenapine	A03CA06	Bevonium And Psycholeptics
N06AF02	Nialamide	N05BA14	Pinazepam	N05AA04	Acepromazine	N05AH06	Clotiapine	A03CA07	Ambutonium And Psycholeptics
N06AF03	Phenelzine	N05BA15	Camazepam	N05AA05	Triflupromazine	N05AL01	Sulpiride	A03CA08	Diphemanil And Psycholeptics
N06AF04	Tranylcypromine	N05BA16	Nordazepam	N05AA06	Cyamemazine	N05AL02	Sultopride	A03CA09	Pipenzolate And Psycholeptics
N06AF05	Iproniazide	N05BA17	Fludiazepam	N05AA07	Chlorproethazine	N05AL03	Tiapride	A03CA30	Emepronium And Psycholeptics
N06AF06	Iproclozide	N05BA18	Ethyl Loflazepate	N05AB01	Dixyrazine	N05AL04	Remoxipride	A03CA34	Propantheline And Psycholeptics
N06AG02	Moclobemide	N05BA19	Etizolam	N05AB02	Fluphenazine	N05AL05	Amisulpride	C01DA70	Organic Nitrates In Combination With Psycholeptics
N06AG03	Toloxatone	N05BA21	Clotiazepam	N05AB03	Perphenazine	N05AL06	Veralipride	N06CA01	Amitriptyline And Psycholeptics
N06AX01	Oxitriptan	N05BA22	Cloxazolam	N05AB04	Prochlorperazine	N05AL07	Levosulpiride	N02BA71	Acetylsalicylic Acid, Combinations With Psycholeptics
N06AX02	Tryptophan	N05BA23	Tofisopam	N05AB05	Thiopropazate	N05AN01	Lithium		
N06AX03	Mianserin	N05BA56	Lorazepam, Comb.	N05AB06	Trifluoperazine	N05AX07	Prothipendyl		
N06AX04	Nomifensine	N05CD01	Flurazepam	N05AB07	Acetophenazine	N05AX08	Risperidone		
N06AX05	Trazodone	N05CD02	Nitrazepam	N05AB08	Thioproperazine	N05AX10	Mosapramine		
N06AX06	Nefazodone	N05CD03	Flunitrazepam	N05AB09	Butaperazine	N05AX11	Zotepine		
N06AX07	Minaprine	N05CD04	Estazolam	N05AB10	Perazine	N05AX12	Aripiprazole		

Abbreviations: ATC, Anatomical Therapeutic Chemical Classification System.

Supplementary Table 2. Psychotropic drugs with at least one dispensation overlapping the pregnancy*

		Mothers treated with antidepressant during pregnancy N=3,982		Mothers treated with Other psychotropic during pregnancy N=1,626	
**ATC**	**Name**	**N**	**%**	**N**	**%**
N06AB06	Sertraline	1326	33.3	73	4.5
N06AB04	Citalopram	1122	28.2	89	5.5
N06AB03	Fluoxetine	639	16	47	2.9
N06AX16	Venlafaxine	405	10.2	19	1.2
N05CF01	Zopiclone	260	6.5	251	15.4
N06AB05	Paroxetine	251	6.3	21	1.3
N05CF02	Zolpidem	242	6.1	262	16.1
N06AB10	Escitalopram	236	5.9	30	1.8
N06AX11	Mirtazapine	218	5.5	21	1.3
N05BA04	Oxazepam	213	5.3	185	11.4
N06AA09	Amitriptyline	187	4.7	14	0.9
N06AA04	Clomipramine	136	3.4	7	0.4
N05BA01	Diazepam	132	3.3	135	8.3
N05BA12	Alprazolam	119	3	70	4.3
N06AX21	Duloxetine	88	2.2	5	0.3
N05AB01	Dixyrazine	68	1.7	442	27.2
N06AX03	Mianserin	58	1.5	0	0
N03AX09	Lamotrigine	53	1.3	190	11.7
N05AH03	Olanzapine	36	0.9	40	2.5
N05CD02	Nitrazepam	28	0.7	31	1.9
N06AX12	Bupropion	28	0.7	3	0.2
N05AA02	Flunitrazepam	25	0.6	22	1.4
N06AX18	Reboxetine	24	0.6	0	0
N05AF01	Flupentixol	23	0.6	14	0.9
N03AG01	Valproic Acid	22	0.6	124	7.6
N05AN01	Lithium	22	0.6	42	2.6
N05AX08	Risperidone	17	0.4	18	1.1
N05CD03	Levomepromazine	15	0.4	32	2
N05AH04	Quetiapine	13	0.3	12	0.7
N06BA04	Methylphenidate	12	0.3	26	1.6
N05AB04	Prochlorperazine	10	0.3	68	4.2
N05AF05	Zuclopenthixol	10	0.3	10	0.6
N05CF03	Zaleplon	10	0.3	18	1.1
N06AA06	Trimipramine	10	0.3	1	0.1
N05AB03	Perphenazine	9	0.2	14	0.9
N06AA10	Nortriptyline	9	0.2	0	0
N05AX12	Aripiprazole	8	0.2	5	0.3
N06AG02	Clozapine	8	0.2	0	0
N03AE01	Clonazepam	7	0.2	19	1.2
N06AB08	Ziprasidone	7	0.2	0	0
N05AE04	Fluvoxamine	5	0.1	2	0.1
N05AD01	Haloperidol	4	0.1	11	0.7
N05AF03	Atomoxetine	3	0.1	3	0.2
N05BA06	Chlorprothixene	3	0.1	3	0.2
N06BA02	Dexamfetamine	3	0.1	1	0.1
N06BA09	Lorazepam	3	0.1	3	0.2
N05AD03	Melperone	2	0.1	0	0
N05AH02	Moclobemide	2	0.1	6	0.4
N05CD05	Triazolam	2	0.1	1	0.1
N06BA01	Amfetamine	2	0.1	8	0.5
N05AB02	Fluphenazine	0	0	1	0.1
N05BA09	Clobazam	0	0	5	0.3
N06BA07	Modafinil	0	0	1	0.1

Abbreviations: ATC, Anatomical Therapeutic Chemical Classification System.

* The sample consists of the mothers with at least two dispensations overlapping pregnancy with either antidepressants (N=3 982) or other psychotropic drugs (N=1 626). The table presents the number of mothers with at least one dispensation overlapping pregnancy with the specific psychotropic drugs considered in the study (see Supplementary Table 1). Any drug listed in Supplementary Table 1 not in this table list was not observed in the sample.

Supplementary Figure 1. Antidepressant dispensations around pregnancy

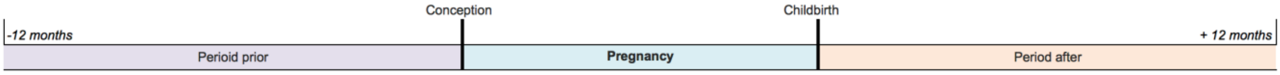

12 months prior pregnancy

	All	No antidepressant overlapping pregnancy
**N**	8,087	2,749
**%**	4.52	1.53

During pregnancy

	All	1 dispensation	>1 dispensation
**N**	6,361	2,379	3,982
**%**	3.55	1.33	2.22

12 months after pregnancy

	All	No antidepressant overlapping pregnancy
**N**	7,622	4,126
**%**	4.26	2.31

Supplementary Figure 2. Medication exposure definitions*

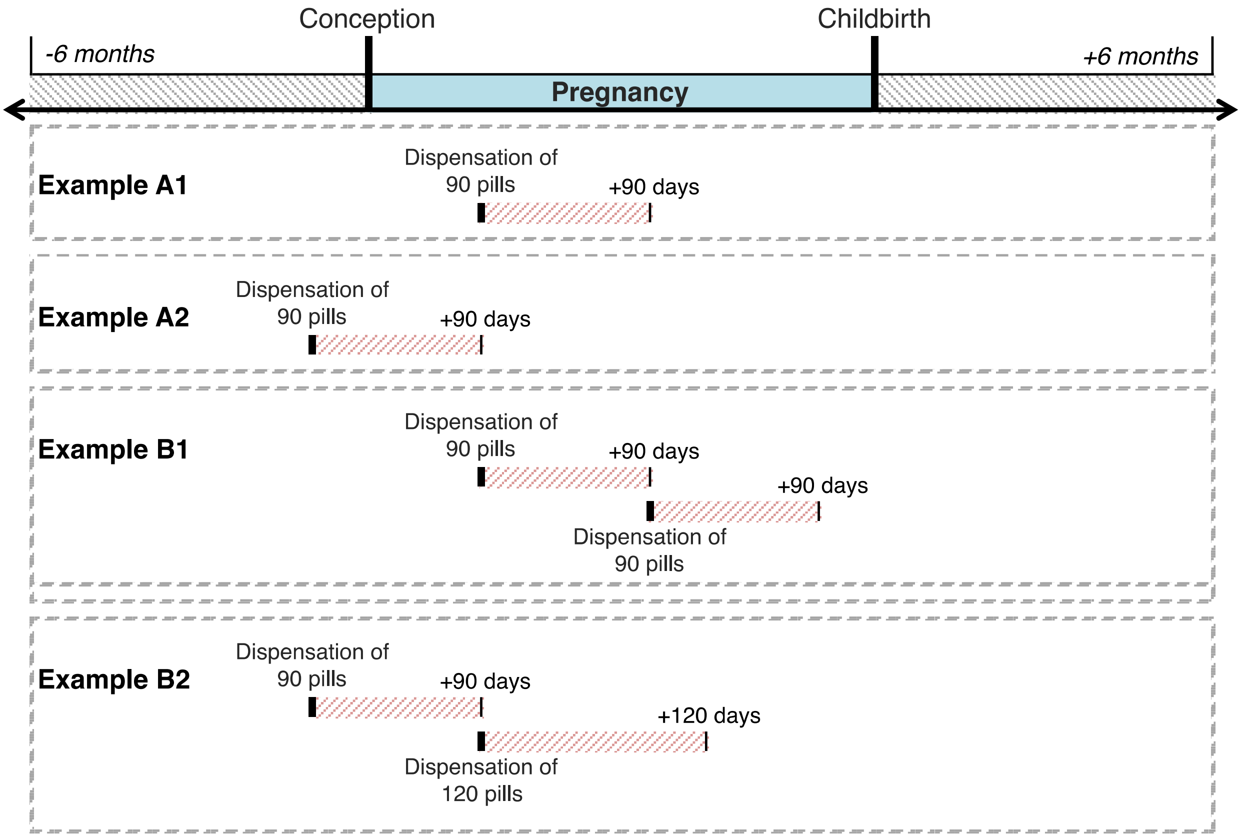

* Exposure to medication during pregnancy was established in both parents using dispensation dates, number of pills dispensed, and the DDD - the assumed average maintenance dose per day for a drug used for its main indication in adults, provided by the World Health Organization (WHO). For antidepressants, medication was assumed to cover the time from the dispensation date until an end date based on number of pills dispensed, assuming one pill was consumed per day. For any other psychotropic drug, the medication period was calculated based on the dispensation dates and the number of pills dispensed divided by the medication-specific DDD. Exposed children were those that born to a mother with either 1) only a single dispensation with a medication period overlapping pregnancy, beginning either during pregnancy (example A1), or prior pregnancy (example A2), or 2) at least two dispensations with medication periods overlapping pregnancy (example B1 and example B2).

Supplementary Table 3. Mental illness sub-groups and corresponding diagnosis codes*

Mental illness subgroup	ICD-10 codes	ICD-9 codes	ICD-8 codes
**Any mental illness**	F00*-F99*	295*-315* (not including: 302, 302.*, 305, 305.*, 307, 307.*, 309, 309.*)	295*-315* (not including: 302, 302.*, 305, 305.*, 307, 307.*, 309, 309.*)
**Depression**	F32*, F33*, F34.1*, F34.8*, F34.9*, F43.21*	296.2*, 296.3*, 296.82, 300.4*, 301.12*, 309.1*, 311*	298.0*, 300.4*
**Anxiety disorders**	F40*, F41*	300.0*, 300.2*	300.0*, 300.2*
**Substance use disorders**	F10*, F11*, F12*, F13*, F14*, F15, F16*, F17*, F18*, F19*	303*, 304*, 305*	303*, 304*
**Bipolar disorder**	F30*, F31*, F34.0*	296.0*, 296.1*, 296.4*, 296.5*, 296.6*, 296.7*, 296.80, 296.81, 296.89, 298B	296.*, 298.1*
**Compulsive disorder**	F42*	300.3	300.3
**ADHD**	F90*	314*	NA
**ASD**	F84.0, F84.1, F84.5, F84.8, F84.9	299B, 299W, 299X	NA
**Schizophrenia**	F20*, F22*, F23*, F24*, F25*, F28*, F29*	295*, 297*, 298*, 298C, 298E, 298W, 298X	295*, 297*, 298.2*, 298.3*, 298.9*, 299*
**Intellectual disability**	F70*-F79*	317*-319*	310*-315*
**Affective disorder**	F38*, F39*	NA	NA

Abbreviations: ICD, International Classification of Diseases. ADHD, attention deficit hyperactivity disorder. ASD, autism spectrum disorder. NA, not available.

*Note: * at the end of a diagnosis code indicates all sub-categories.

Supplementary Figure 3. Relative risks of ASD in offspring of mothers with one, or at least two medication dispensations overlapping pregnancy*

	At least two dispensations		One single dispensation	
	Any antidepressant N=3,982	Other psychotropics[1] N=1,626	Any antidepressant N=2,379	Other psychotropics N=1,535
	Relative risk (95% CI)	Relative risk (95% CI)	Relative risk (95% CI)	Relative risk (95% CI)
Model 1[2]	2.46 (1.97-3.05)	2.21 (1.57-3.12)	1.55 (1.09-2.20)	1.27 (0.80-2.03)
Model 2[3]	2.10 (1.66-2.65)	2.11 (1.48-3.01)	1.43 (1.01-2.04)	1.31 (0.82-2.09)
Model 3[4]	1.42 (1.12-1.81)	1.75 (1.22-2.50)	1.08 (0.76-1.54)	1.20 (0.75-1.91)
Model 4[5]	1.23 (0.96-1.57)	1.43 (0.99-2.05)	0.98 (0.69-1.40)	1.08 (0.68-1.72)

Abbreviations: ASD, autism spectrum disorder. N, number of births to treated mothers. SSRI, selective serotonin re-uptake inhibitor. CI, confidence interval.

* The sample consists of 179 007 children born during 2006 and 2007, of which 1 641 had been diagnosed with ASD. The figure presents relative risks of ASD and two-sided 95% confidence intervals in children of mothers with either a single drug dispensation with a medication period overlapping pregnancy (Supplementary Figure 1 example A1 and example A2), or at least two drug dispensations with medication periods overlapping the pregnancy, compared with unexposed children (Supplementary Figure 1 example B1 and example B2).

[1] Mothers not using antidepressant, but other psychotropic medication during pregnancy.

[2] Analyses not adjusted for covariates.

[3] Analyses adjusted for birthdate, maternal and paternal age, the father's psychotropic medication overlapping the pregnancy, and the mother's one-time dispensations of antidepressant and/or other psychotropic medication that overlapped the pregnancy.

[4] Analyses adjusted for the factors listed in [3], and for any diagnosis of depression in the mother's lifetime (yes/no) (see Supplement Table S3 for specific diagnosis codes).

[5] Analyses adjusted for the factors listed in [3], and for any diagnosis of specific psychiatric disorder subgroups in either the mother and/or father's life time (yes/no), including depression, anxiety disorders, substance use disorder, bipolar disorder, compulsive disorder, attention deficit hyperactive disorder (ADHD), autism spectrum disorder, intellectual disability, schizophrenia, and 'other psychiatric diagnosis' (see Supplement Table 3 for specific diagnosis codes).

Supplementary Figure 4. Plotted Schoenfeldt residuals for each covariate with 4 degrees of freedom

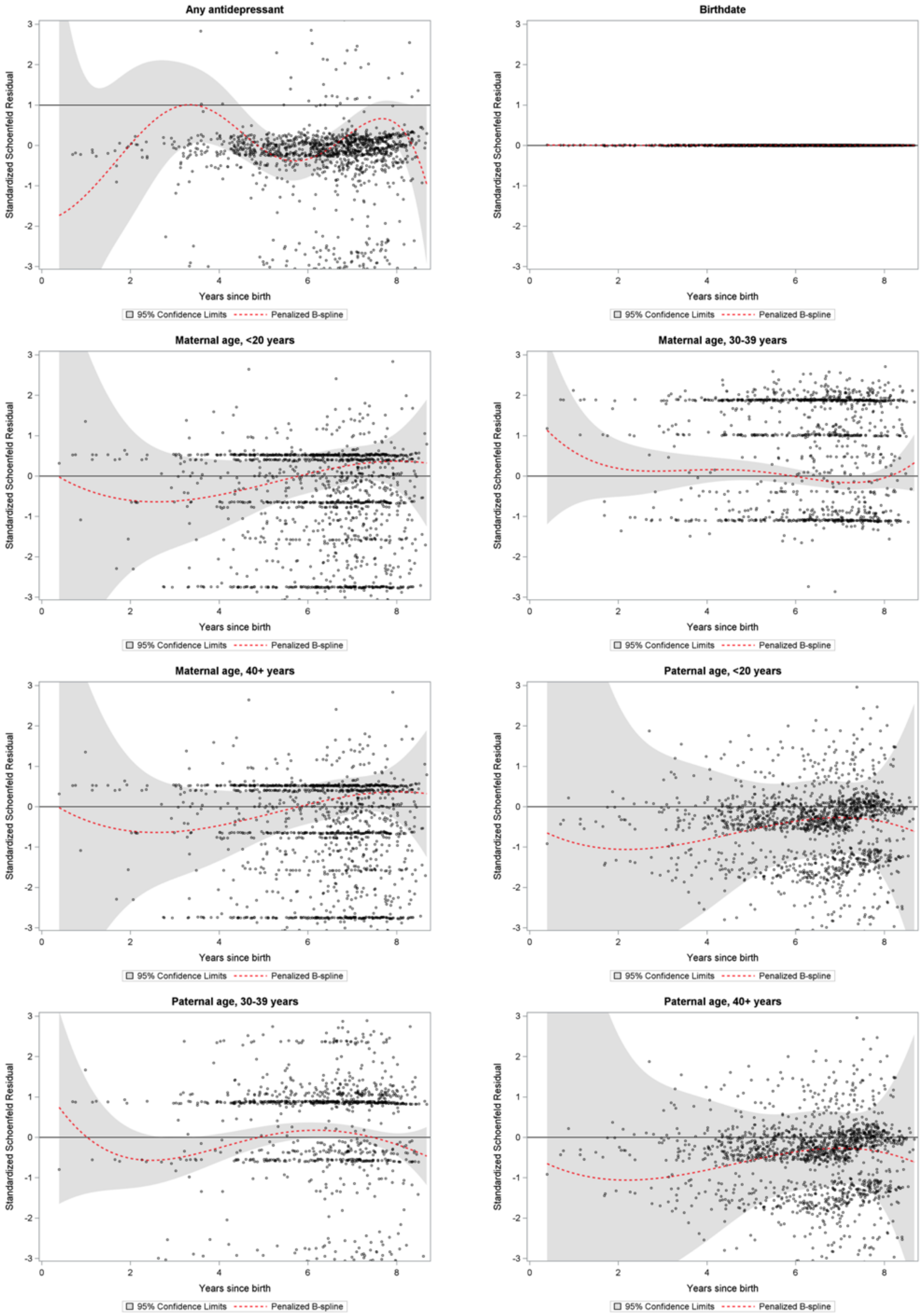

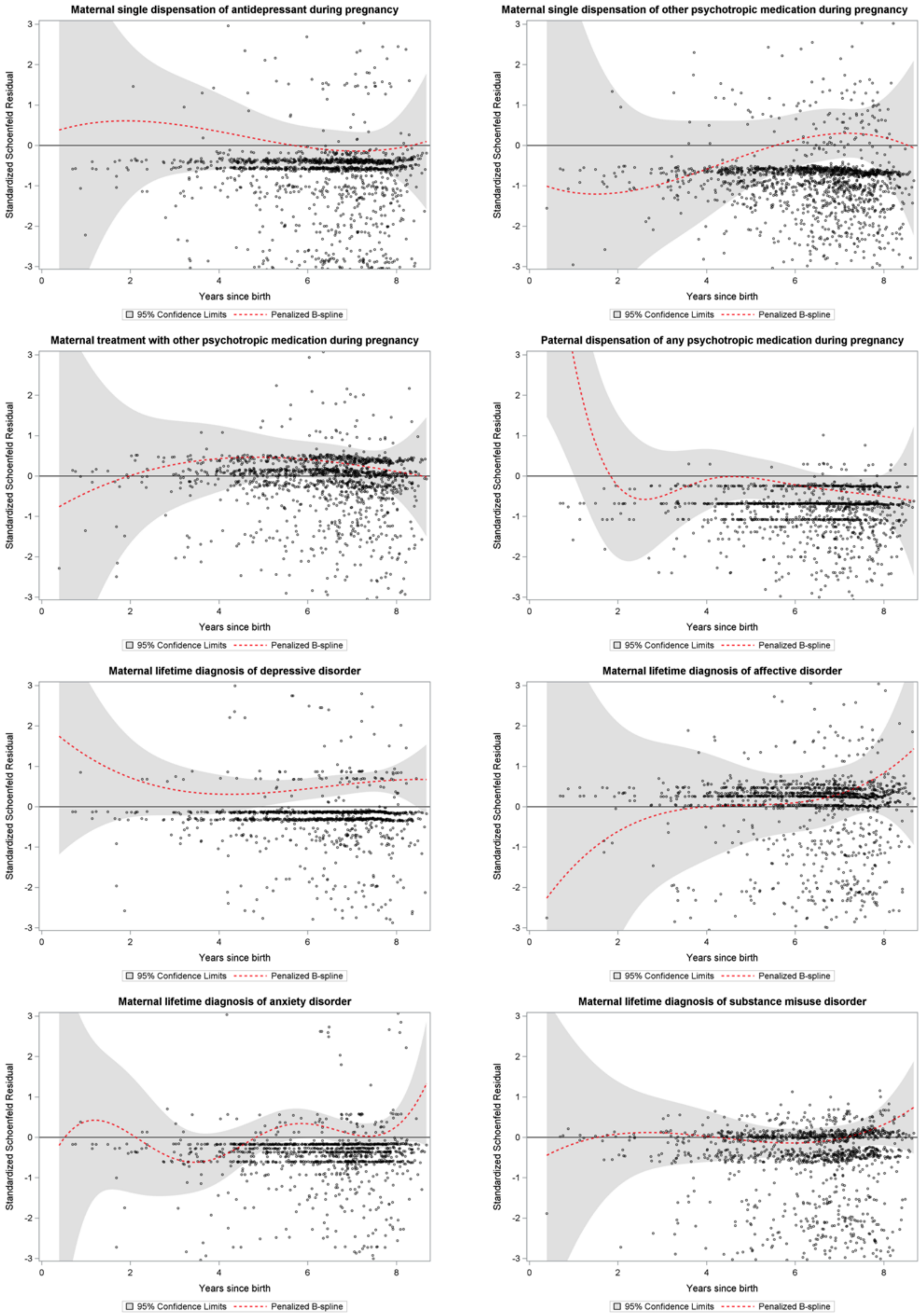
Maternal single dispensation of antidepressant during pregnancy
Maternal single dispensation of other psychotropic medication during pregnancy
Maternal treatment with other psychotropic medication during pregnancy
Paternal dispensation of any psychotropic medication during pregnancy
Maternal lifetime diagnosis of depressive disorder
Maternal lifetime diagnosis of affective disorder
Maternal lifetime diagnosis of anxiety disorder
Maternal lifetime diagnosis of substance misuse disorder
Standardized Schoenfeld Residual
Years since birth
95% Confidence Limits
Penalized B-spline

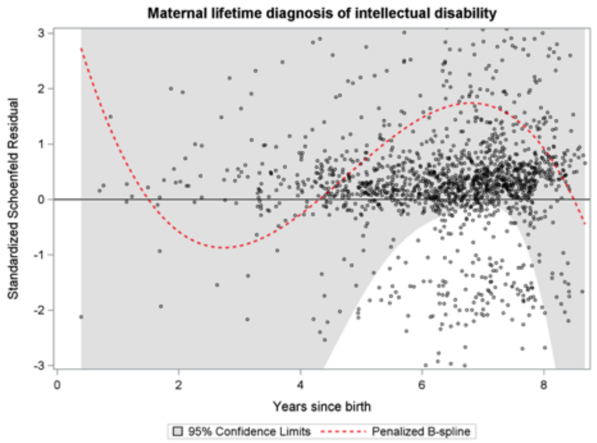
Maternal lifetime diagnosis of intellectual disability
Standardized Schoenfeld Residual
Years since birth
95% Confidence Limits
Penalized B-spline

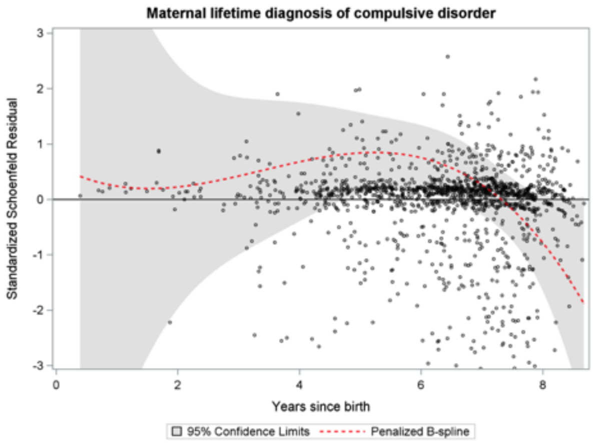
Maternal lifetime diagnosis of compulsive disorder
Standardized Schoenfeld Residual
Years since birth
95% Confidence Limits
Penalized B-spline

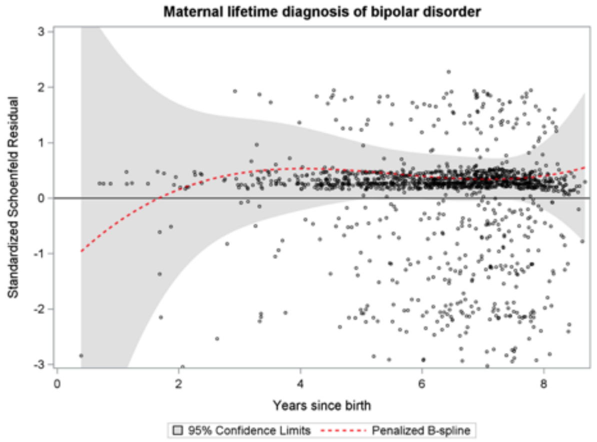
Maternal lifetime diagnosis of bipolar disorder
Standardized Schoenfeld Residual
Years since birth
95% Confidence Limits
Penalized B-spline

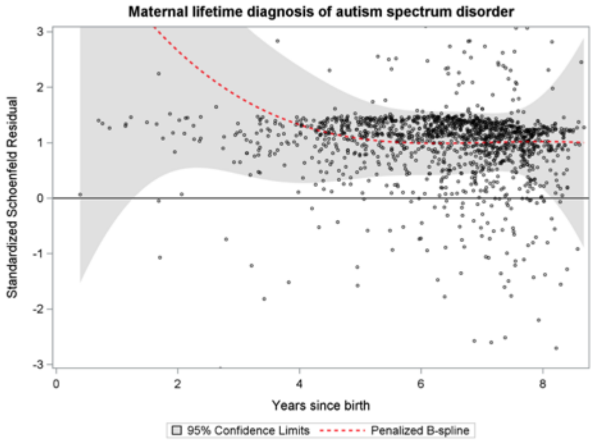
Maternal lifetime diagnosis of autism spectrum disorder
Standardized Schoenfeld Residual
Years since birth
95% Confidence Limits
Penalized B-spline

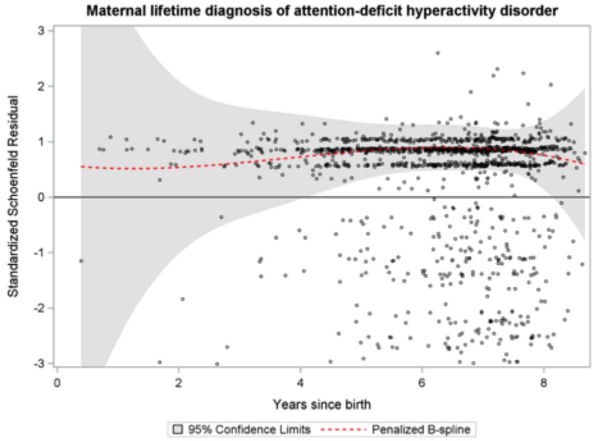
Maternal lifetime diagnosis of attention-deficit hyperactivity disorder
Standardized Schoenfeld Residual
Years since birth
95% Confidence Limits
Penalized B-spline

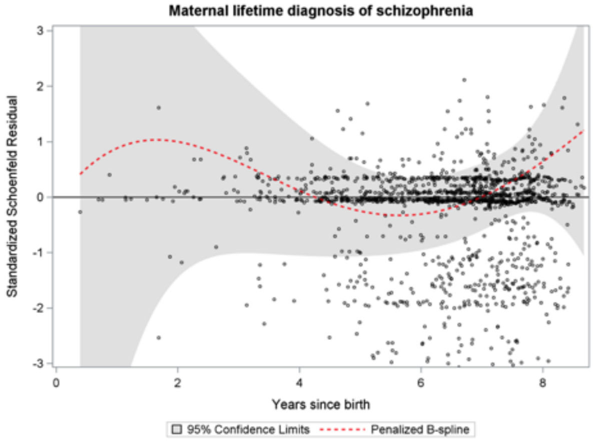
Maternal lifetime diagnosis of schizophrenia
Standardized Schoenfeld Residual
Years since birth
95% Confidence Limits
Penalized B-spline

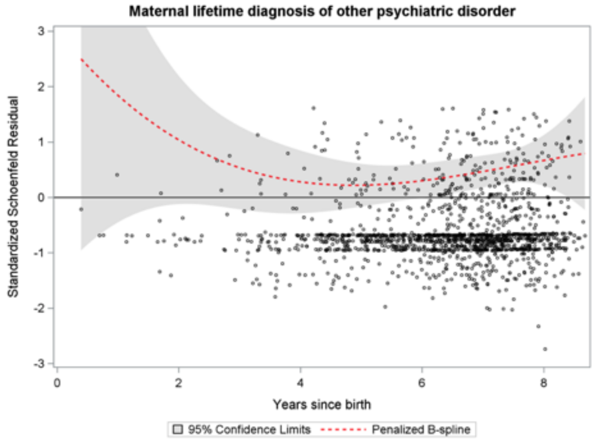
Maternal lifetime diagnosis of other psychiatric disorder
Standardized Schoenfeld Residual
Years since birth
95% Confidence Limits
Penalized B-spline

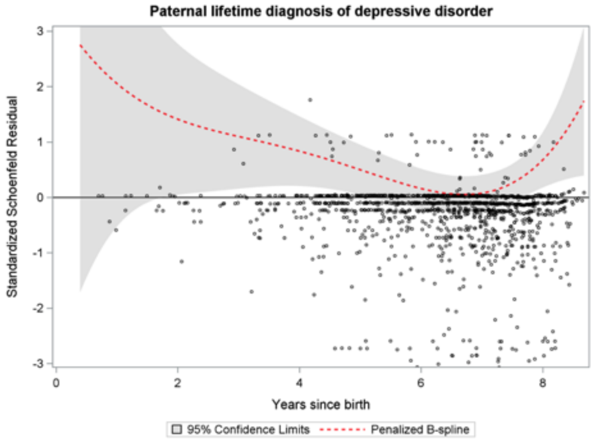
Paternal lifetime diagnosis of depressive disorder
Standardized Schoenfeld Residual
Years since birth
95% Confidence Limits
Penalized B-spline

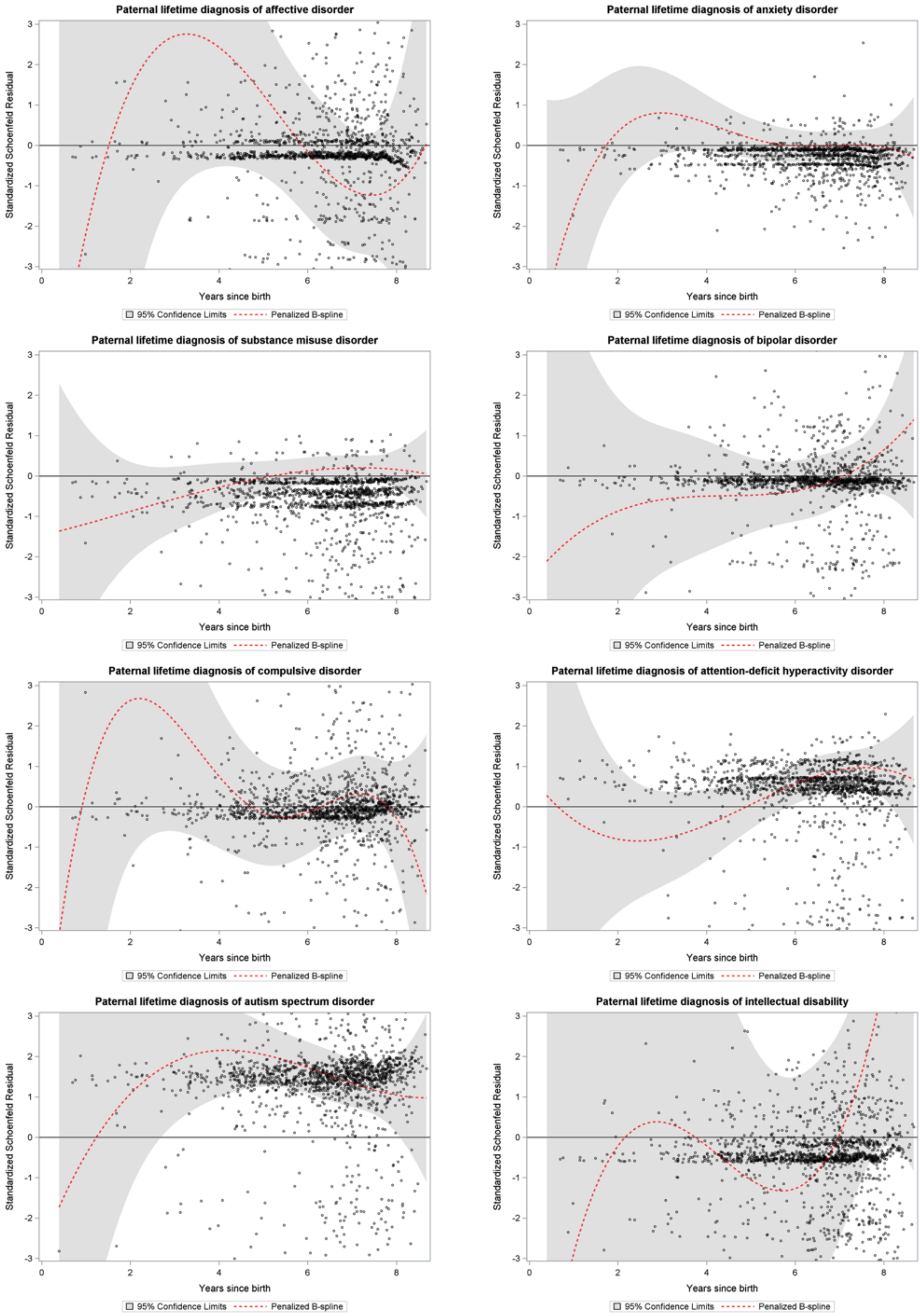

Paternal lifetime diagnosis of affective disorder
Paternal lifetime diagnosis of anxiety disorder
Paternal lifetime diagnosis of substance misuse disorder
Paternal lifetime diagnosis of bipolar disorder
Paternal lifetime diagnosis of compulsive disorder
Paternal lifetime diagnosis of attention-deficit hyperactivity disorder
Paternal lifetime diagnosis of autism spectrum disorder
Paternal lifetime diagnosis of intellectual disability
Standardized Schoenfeld Residual
Years since birth
95% Confidence Limits
Penalized B-spline

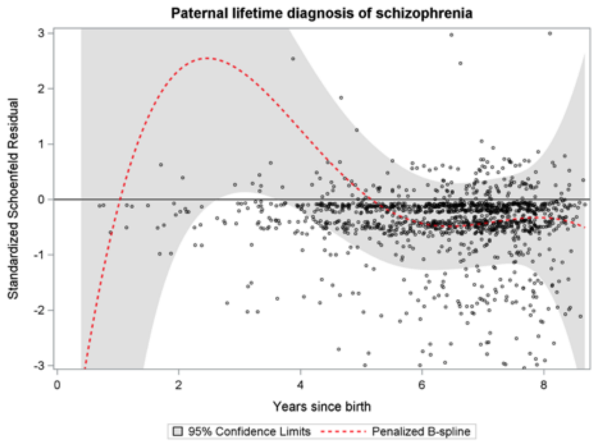

Paternal lifetime diagnosis of schizophrenia
Standardized Schoenfeld Residual
Years since birth
95% Confidence Limits
Penalized B-spline

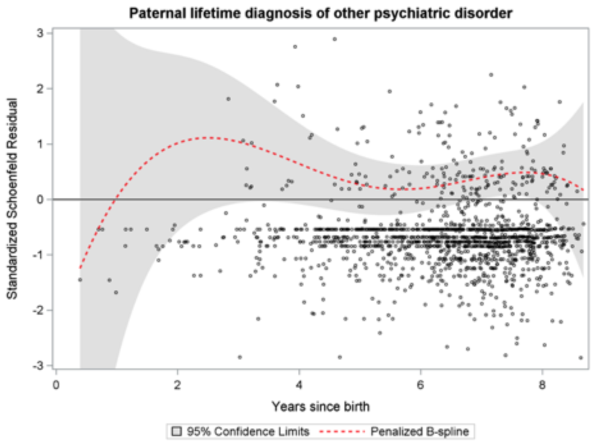

Paternal lifetime diagnosis of other psychiatric disorder
Standardized Schoenfeld Residual
Years since birth
95% Confidence Limits
Penalized B-spline

Supplementary Figure 5. Relative risks of ASD in offspring of mother treated with any antidepressant, or specifically SSRI antidepressants, non-SSRI antidepressants, or non-antidepressant psychotropic drugs*

	Any antidepressant N=3,982	SSRI antidepressants N=3,178	Other antidepressants N=804	Other psychotropics N=1,626
	Relative risk (95% CI)	Relative risk (95% CI)	Relative risk (95% CI)	Relative risk (95% CI)
Model 1[1]	2.46 (1.97-3.05)	2.50 (1.96-3.18)	2.28 (1.39-3.73)	2.21 (1.57-3.12)
Model 2[2]	2.10 (1.66-2.65)	2.17 (1.68-2.78)	1.88 (1.11-3.05)	2.11 (1.48-3.01)
Model 3[3]	1.42 (1.12-1.81)	1.47 (1.13-1.91)	1.25 (0.75-2.08)	1.75 (1.22-2.50)
Model 4[4]	1.23 (0.96-1.57)	1.30 (0.99-1.69)	0.98 (0.59-1.64)	1.43 (0.99-2.05)

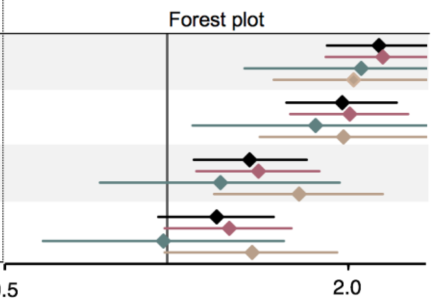

Abbreviations: ASD, autism spectrum disorder. N, number of births to treated mothers. SSRI, selective serotonin re-uptake inhibitor. CI, confidence interval.

* The sample consists of 179 007 children born during 2006 and 2007, of which 1 641 had been diagnosed with ASD. The figure presents relative risks of ASD and two-sided 95% confidence intervals in children of mothers with at least two dispensations of antidepressants or other psychotropic medications overlapping the pregnancy, compared with unexposed children.

[1] Analyses not adjusted for covariates.

[2] Analyses adjusted for birthdate, maternal and paternal age, the father's psychotropic medication overlapping the pregnancy, and the mother's one-time dispensations of antidepressant and/or other psychotropic medication that overlapped the pregnancy.

[3] Analyses adjusted for the factors listed in [3], and for any diagnosis of depression in the mother's lifetime (yes/no) (see Supplement Table S3 for specific diagnosis codes).

[4] Analyses adjusted for the factors listed in [3], and for any diagnosis of specific psychiatric disorder subgroups in either the mother and/or father's life time (yes/no), including depression, anxiety disorders, substance use disorder, bipolar disorder, compulsive disorder, attention deficit hyperactive disorder (ADHD), autism spectrum disorder, intellectual disability, schizophrenia, and 'other psychiatric diagnosis' (see Supplement Table 3 for specific diagnosis codes).

Supplementary Figure 6. Relative risks of ASD from bootstrapped analyses*

	Any antidepressant N=3,982	SSRI antidepressants N=3,178	Other antidepressants N=804	Other psychotropics N=1,626
	Relative risk (95% CI)	Relative risk (95% CI)	Relative risk (95% CI)	Relative risk (95% CI)
Model 1[1]	2.46 (1.99-2.95)	2.50 (1.96-3.11)	2.28 (1.28-3.42)	2.21 (1.49-3.06)
Model 2[2]	2.10 (1.70-2.53)	2.17 (1.62-2.82)	1.84 (1.02-3.03)	2.11 (1.33-2.84)
Model 3[3]	1.42 (1.03-1.82)	1.47 (1.09-1.88)	1.25 (0.56-2.05)	1.75 (1.18-2.38)
Model 4[4]	1.23 (1.01-1.53)	1.30 (1.08-1.68)	0.98 (0.50-1.46)	1.43 (0.86-2.14)

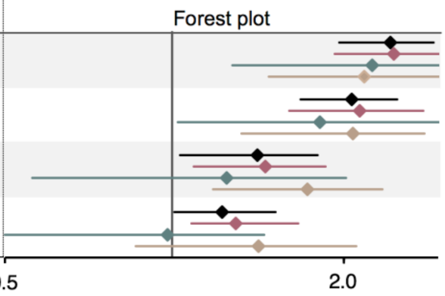

Abbreviations: ASD, autism spectrum disorder. N, number of births to treated mothers. SSRI, selective serotonin re-uptake inhibitor. CI, confidence interval.

* The sample consists of 179 007 children born during 2006 and 2007, of which 1 641 had been diagnosed with ASD. Bootstrapping was performed with 100 repetitions. The figure presents relative risks of ASD and two-sided 95% confidence intervals in children of mothers with at least two dispensations of antidepressants or other psychotropic medications overlapping the pregnancy, compared with unexposed children.

[1] Analyses not adjusted for covariates.

[2] Analyses adjusted for birthdate, maternal and paternal age, the father's psychotropic medication overlapping the pregnancy, and the mother's one-time dispensations of antidepressant and/or other psychotropic medication that overlapped the pregnancy.

[3] Analyses adjusted for the factors listed in [3], and for any diagnosis of depression in the mother's lifetime (yes/no) (see Supplement Table S3 for specific diagnosis codes).

[4] Analyses adjusted for the factors listed in [3], and for any diagnosis of specific psychiatric disorder subgroups in either the mother and/or father's life time (yes/no), including depression, anxiety disorders, substance use disorder, bipolar disorder, compulsive disorder, attention deficit hyperactive disorder (ADHD), autism spectrum disorder, intellectual disability, schizophrenia, and 'other psychiatric diagnosis' (see Supplement Table 3 for specific diagnosis codes).

Supplementary Figure 7. Relative risks of autistic disorder*

	Any antidepressant N=3,982	SSRI antidepressants N=3,178	Other antidepressants N=804	Other psychotropics N=1,626
	Relative risk (95% CI)	Relative risk (95% CI)	Relative risk (95% CI)	Relative risk (95% CI)
Model 1[1]	1.90 (1.39-2.59)	1.91 (1.35-2.71)	1.83 (0.91-3.66)	2.20 (1.41-3.43)
Model 2[2]	1.65 (1.19-2.30)	1.69 (1.18-2.42)	1.50 (0.74-3.05)	2.01 (1.27-3.17)
Model 3[3]	1.15 (0.82-1.62)	1.18 (0.81-1.71)	1.05 (0.51-2.14)	1.69 (1.06-2.67)
Model 4[4]	1.01 (0.71-1.42)	1.04 (0.72-1.52)	0.88 (0.43-1.80)	1.36 (0.86-2.17)

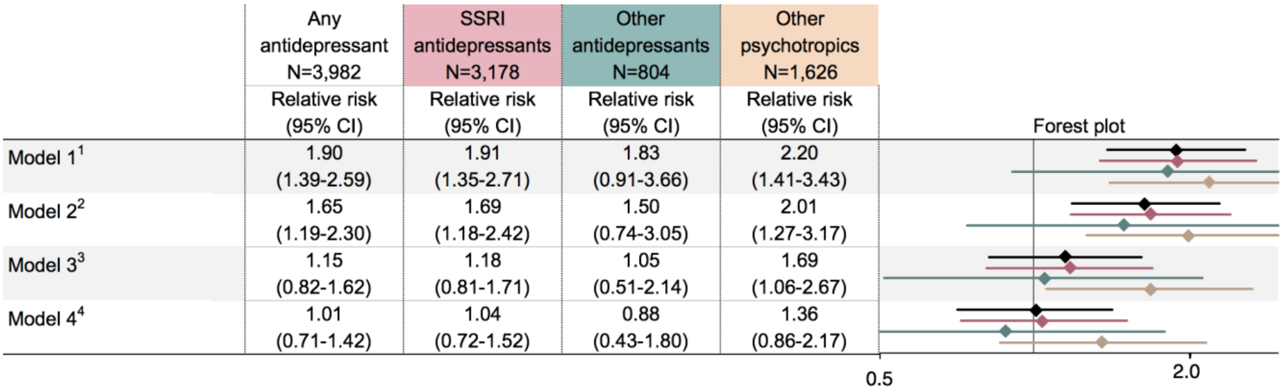

Abbreviations: N, number of births to treated mothers. SSRI, selective serotonin re-uptake inhibitor. CI, confidence interval.

* The sample consists of 179 007 children born during 2006 and 2007, of which 1 004 had been diagnosed with autistic disorder (ICD-10: F84.0). The figure presents relative risks of ASD and two-sided 95% confidence intervals in children of mothers with at least two dispensations of antidepressants or other psychotropic medications overlapping the pregnancy, compared with unexposed children.

[1] Analyses not adjusted for covariates.

[2] Analyses adjusted for birthdate, maternal and paternal age, the father's psychotropic medication overlapping the pregnancy, and the mother's one-time dispensations of antidepressant and/or other psychotropic medication that overlapped the pregnancy.

[3] Analyses adjusted for the factors listed in [3], and for any diagnosis of depression in the mother's lifetime (yes/no) (see Supplement Table S3 for specific diagnosis codes).

[4] Analyses adjusted for the factors listed in [3], and for any diagnosis of specific psychiatric disorder subgroups in either the mother and/or father's life time (yes/no), including depression, anxiety disorders, substance use disorder, bipolar disorder, compulsive disorder, attention deficit hyperactive disorder (ADHD), autism spectrum disorder, intellectual disability, schizophrenia, and 'other psychiatric diagnosis' (see Supplement Table 3 for specific diagnosis codes).

Supplementary Figure 8. Sex-combined and sex-specific relative risks of ASD*

	Any antidepressant N=3,982 Relative risk (95% CI)		
	Combined	Males	Females
Model 1[1]	2.46 (1.97-3.05)	2.43 (1.88-3.13)	2.56 (1.66-3.93)
Model 2[2]	2.10 (1.66-2.65)	2.07 (1.58-2.72)	2.20 (1.40-3.46)
Model 3[3]	1.42 (1.12-1.81)	1.43 (1.08-1.90)	1.40 (0.87-2.25)
Model 4[4]	1.23 (0.96-1.57)	1.28 (0.96-1.70)	1.15 (0.71-1.86)

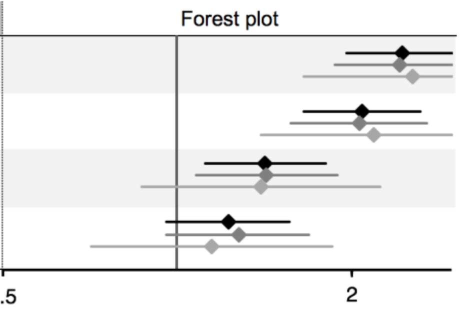

Abbreviations: ASD, autism spectrum disorder. N, number of births to treated mothers. CI, confidence interval.

* The sample consists of 179 007 children born during 2006 and 2007, of which 90 090 (51.5%) were males and 84 935 (48.5%) females. There were 1 641 children diagnosed with ASD, of which 1 233 (75.1%) where male and 408 (24.9%) female. The figure presents relative risks of ASD and two-sided 95% confidence intervals in children of mothers with at least two dispensations of antidepressants or other psychotropic medications overlapping the pregnancy, compared with unexposed children.

[1] Analyses not adjusted for covariates.

[2] Analyses adjusted for birthdate, maternal and paternal age, the father's psychotropic medication overlapping the pregnancy, and the mother's one-time dispensations of antidepressant and/or other psychotropic medication that overlapped the pregnancy.

[3] Analyses adjusted for the factors listed in [3], and for any diagnosis of depression in the mother's lifetime (yes/no) (see Supplement Table S3 for specific diagnosis codes).

[4] Analyses adjusted for the factors listed in [3], and for any diagnosis of specific psychiatric disorder subgroups in either the mother and/or father's life time (yes/no), including depression, anxiety disorders, substance use disorder, bipolar disorder, compulsive disorder, attention deficit hyperactive disorder (ADHD), autism spectrum disorder, intellectual disability, schizophrenia, and 'other psychiatric diagnosis' (see Supplement Table 3 for specific diagnosis codes).

Supplementary Figure 9. Analysis additionally adjusted for socioeconomic status as parental education level*

	Full sample N=179,007 Relative risk (95% CI)	Clinical sub-sample[1] N=18,551 Relative risk (95% CI)
Model 1[2]	2.46 (1.97-3.05)	1.30 (0.99-1.71)
Model 2[3]	2.10 (1.66-2.65)	1.17 (0.88-1.57)
Model 3[4]	1.42 (1.12-1.81)	1.15 (0.86-1.53)
Model 4[5]	1.23 (0.96-1.57)	1.07 (0.80-1.43)
Model 5[6]	1.23 (0.96-1.57)	1.09 (0.81-1.46)

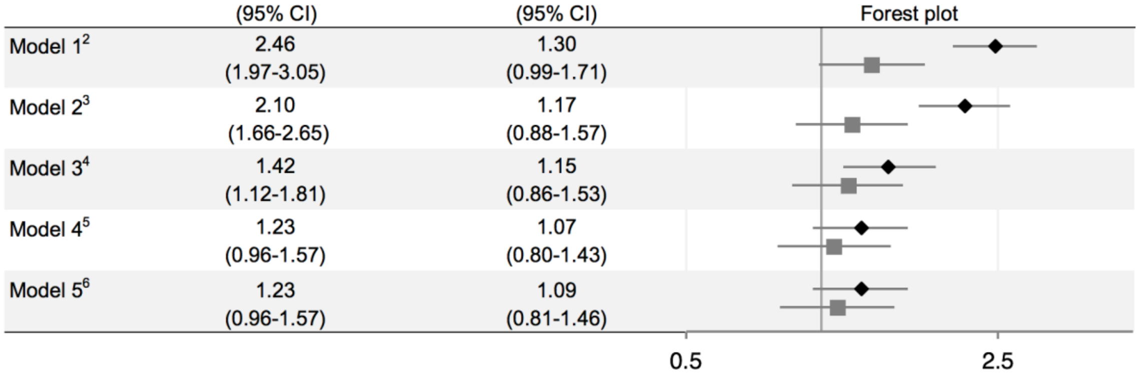

Abbreviations: N, number of children. CI, confidence interval.

* The sample consists of 179,007 children born during 2006 and 2007, of which 1,641 had been diagnosed with ASD. The figure presents relative risks of ASD and two-sided 95% confidence intervals in children of mothers with at least two dispensations of antidepressant drugs overlapping the pregnancy, compared with unexposed children.

[1] A clinically relevant sub-sample consisting of 18,551 children, of which 361 had been diagnosed with ASD. All mothers, both medicated and non-medicated, had at least one diagnosis of depression or an anxiety disorder in their lifetime (Table S3). Thereby, the offspring of mothers with medication during pregnancy is contrasted with offspring of mothers that may share similar underlying factors.

[2] Analyses not adjusted for covariates.

[3] Analyses adjusted for birthdate, maternal and paternal age, the father's psychotropic medication that overlapped the pregnancy, and the mother's one-time dispensations of psychotropic medication that overlapped the pregnancy.

[4] Analyses adjusted for the factors listed in [3], and for any diagnosis of depression in the mother's lifetime (yes/no) (see Supplement Table S3 for specific diagnosis codes).

[5] Analyses adjusted for the factors listed in [3], and for any diagnosis of specific psychiatric disorder subgroups in either the mother and/or father's life time (yes/no), including depression, anxiety disorders, substance use disorder, bipolar disorder, compulsive disorder, attention deficit hyperactive disorder (ADHD), autism spectrum disorder, intellectual disability, schizophrenia, and 'other psychiatric diagnosis' (see Supplement Table 3 for specific diagnosis codes).

[5] Analyses adjusted for the factors listed in [5], and for socioeconomic status in form of the mothers' and fathers' educational length at childbirth according to: 1) Compulsory school shorter than 9 years, 2) Compulsory school 9 years, 3) Upper secondary school up to 2 years, 4) Upper secondary school 3 years, 5) Post-secondary education less than 3 years, 6) University 3 years, and 7) Doctoral education.

Supplementary Figure 10. Percentage of mothers with at least one lifetime diagnosis in different psychiatric disorder categories*

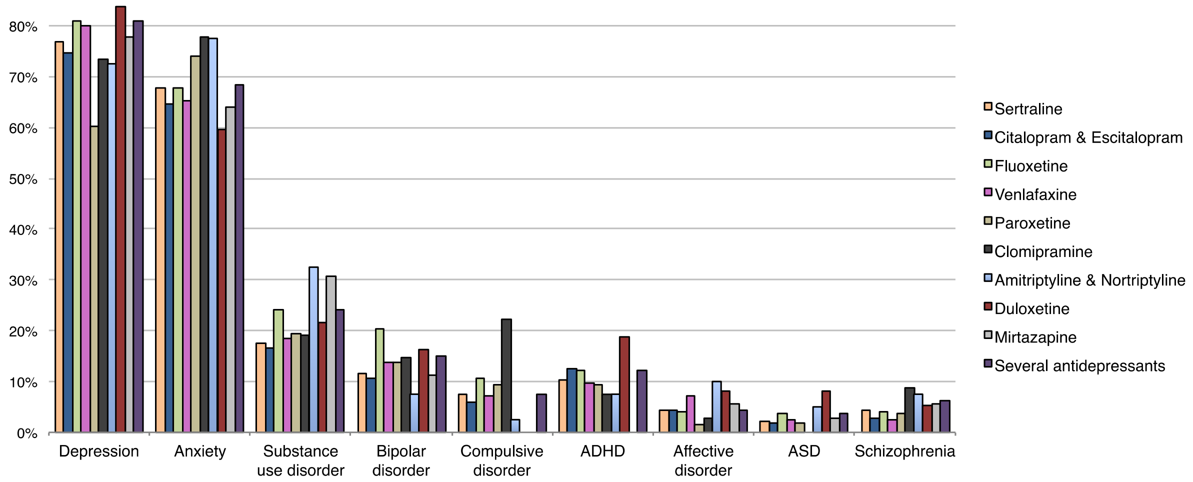

Abbreviations: ADHD, attention deficit hyperactivity disorder, ASD, autism spectrum disorder.

* The sample consists of mothers having at least one lifetime depression or anxiety diagnosis, and at least two dispensations of antidepressants (corresponding to the medicated mothers in Figure 2). The exact diagnosis codes for each category are given in Supplementary Table 3.

Supplementary Figure 11. Relative risks of ASD in offspring of mothers treated with any antidepressant, compared to control children of mothers not using any psychotropic medication but with increasing mean number of psychiatric disorders diagnosed during lifetime

	Antidepressant exposed children		Control children			
Control group mothers' number of lifetime diagnoses within different psychiatric disorder categories	Number of children	Mothers' mean number of lifetime diagnoses within different psychiatric disorder categories	Number of children	Mothers' mean number of lifetime diagnoses within different psychiatric disorder categories	Adjusted Relative risk (95% CI)	Forest plot
At least 1	3,982	1.45	24,285	1.35	1.26 (1.00-1.60)	
At least 2	3,982	1.45	5,839	2.44	0.96 (0.73-1.27)	
At least 3	3,982	1.45	1,894	3.35	0.70 (0.50-0.98)	

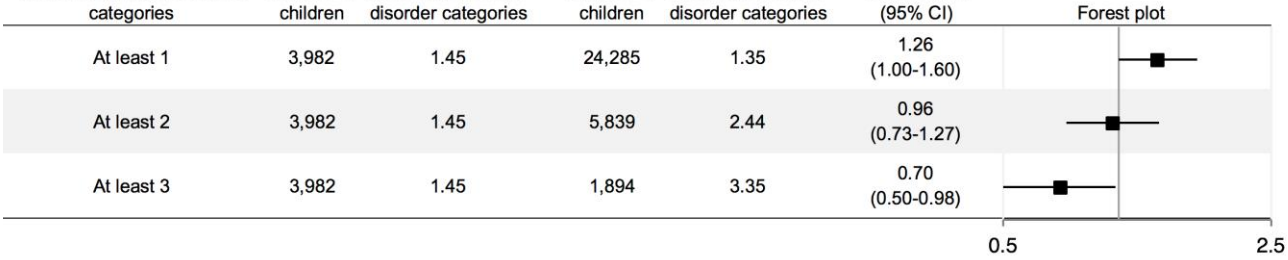

* Children of mothers with at least two dispensations of antidepressants overlapping pregnancy (N=3,982), compared to control children of mothers without any psychotropic medication dispensed with a medication period overlapping pregnancy, but with at least one lifetime diagnosed psychiatric disorder (N=24,285), at least two lifetime diagnosed psychiatric disorders (N=5839), or at least three lifetime diagnosed psychiatric disorders (N=1,894). The psychiatric disorders included 11 categories described in Supplementary Table 3, and included: Depression, anxiety disorder, substance use disorder, bipolar disorder, compulsive disorder, ADHD, autism spectrum disorder, intellectual disability, schizophrenia, and 'other psychiatric diagnosis', each coded as 1 or 0.

Supplementary Figure 12. Relative risks of ASD in offspring of mothers treated with specific antidepressants, compared to control children of mothers not using any psychotropic medication but with increasing mean number of psychiatric disorders diagnosed during lifetime*

	Antidepressant exposed children		Control groups with mothers having at least X number of lifetime diagnoses within different psychiatric disorder categories								
			■ At least 1			● At least 2			◆ At least 3		
Medication group	Number of children	Mothers' mean number of lifetime diagnoses within different psychiatric disorder categories	Number of children	Mothers' mean number of lifetime diagnoses within different psychiatric disorder categories[3]	Adjusted Relative risk (95% CI)	Number of children	Mothers' mean number of lifetime diagnoses within different psychiatric disorder categories	Adjusted Relative risk (95% CI)	Number of children	Mothers' mean number of lifetime diagnoses within different psychiatric disorder categories	Adjusted Relative risk (95% CI)
Sertraline	1,016	1.43	24,285	1.35	1.32 (0.86-2.24)	5,839	2.44	0.99 (0.63-1.55)	1,894	3.35	0.71 (0.43-1.17)
Citalopram & escitalopram	1,097	1.21	24,285	1.35	1.52 (1.03-2.03)	5,839	2.44	1.16 (0.77-1.75	1,894	3.35	0.84 (0.53-1.33)
Fluoxetine	458	1.73	24,285	1.35	1.18 (0.61-2.29)	5,839	2.44	0.91 (0.46-1.79)	1,894	3.35	0.66 (0.33-1.34)
Venlafaxine	266	1.59	24,285	1.35	1.36 (0.61-3.04)	5,839	2.44	1.01 (0.44-2.29)	1,894	3.35	0.74 (0.32-1.72)
Paroxetine	173	1.30	24,285	1.35	1.36 (0.51-3.64)	5,839	2.44	1.02 (0.38-2.78)	1,894	3.35	0.74 (0.27-2.04)
Clomipramine	91	1.76	24,285	1.35	3.36 (1.39-8.13)	5,839	2.44	2.53 (1.03-6-22)	1,894	3.35	1.88 (0.75-4.73)
Amitriptyline & nortriptyline	133	0.81	24,285	1.35	0.47 (0.07-3.31)	5,839	2.44	0.35 (0.05-2.49)	1,894	3.35	0.25 (0.04-1.84)
Duloxetine	52	1.60	24,285	1.35	NA	5,839	2.44	NA	1,894	3.35	NA
Mirtazapine	62	1.34	24,285	1.35	1.55 (0.39-6.29)	5,839	2.44	1.24 (0.30-5.06)	1,894	3.35	0.99 (0.24-4.09)

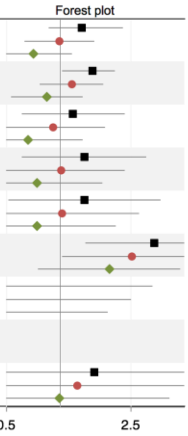

* Children of mothers with at least two dispensations of specific antidepressants overlapping pregnancy, compared to control children of mothers without any psychotropic medication dispensed with a medication period overlapping pregnancy, but with at least one lifetime diagnosed psychiatric disorder (N=24,285; black box in plot), at least two lifetime diagnosed psychiatric disorders (N=5839; red circle in plot), or at least three lifetime diagnosed psychiatric disorders (N=1,894; green diamond in plot). The psychiatric disorders included 11 categories described in Supplementary Table 3, and included: Depression, anxiety disorder, substance use disorder, bipolar disorder, compulsive disorder, ADHD, autism spectrum disorder, intellectual disability, schizophrenia, and 'other psychiatric diagnosis', each coded as 1 or 0.

Supplementary Figure 13. Percentage of medicated mothers with a lifetime psychiatric diagnosis*

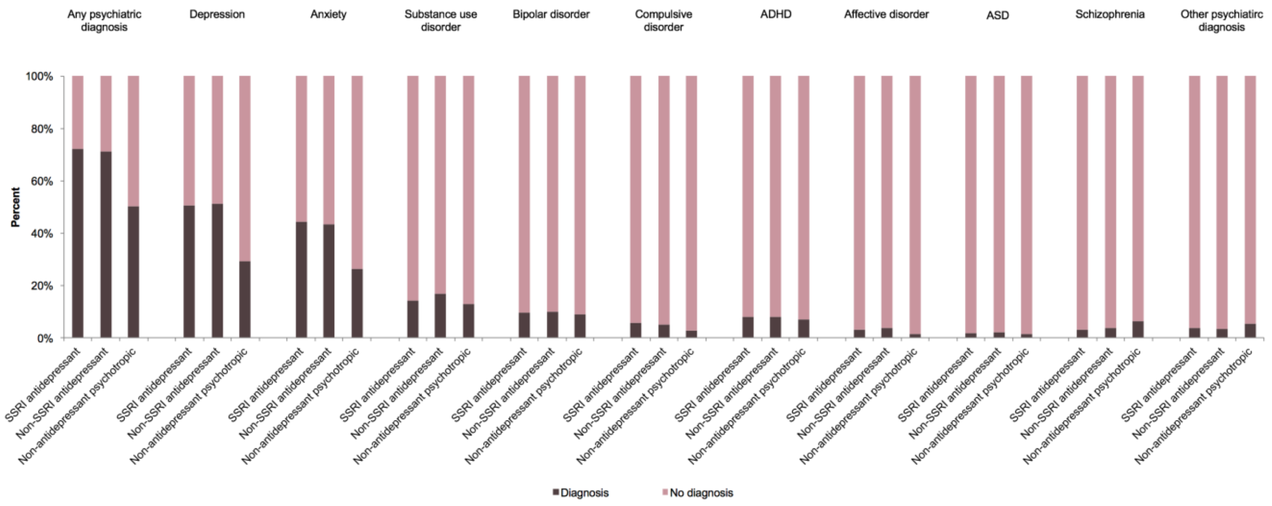

Abbreviations: SSRI, selective serotonin re-uptake inhibitor. ADHD, attention-deficit hyperactive disorder. ASD, autism spectrum disorder.

*The sample consists of the mothers with at least two antidepressant dispensations overlapping the pregnancy (N=3 982) or at least two dispensations of other psychotropic medications overlapping the pregnancy (N=1 626).
